# Natural killer cells kill extracellular *Pseudomonas aeruginosa* using contact-dependent release of granzymes B and H

**DOI:** 10.1371/journal.ppat.1010325

**Published:** 2022-02-24

**Authors:** David D. Feehan, Khusraw Jamil, Maria J. Polyak, Henry Ogbomo, Mark Hasell, Shu Shun LI, Richard F. Xiang, Michael Parkins, Joseph A. Trapani, Joe J. Harrison, Christopher H. Mody

**Affiliations:** 1 Calvin, Phoebe, and Joan Snyder Institute for Chronic Diseases, University of Calgary, Calgary, Alberta, Canada; 2 Department of Microbiology, Immunology, and Infectious Diseases, Cumming School of Medicine, Calgary, Alberta, Canada; 3 Department of Family Medicine, University of Calgary, Calgary, AB T2N 4N1, Canada; 4 Department of Medicine, University of Calgary, Calgary, Alberta, Canada; 5 Cancer Immunology Program, Peter MacCallum Cancer Centre, Melbourne, Victoria, Australia; 6 Sir Peter MacCallum Department of Oncology, University of Melbourne, Parkville, Victoria, Australia; 7 Department of Biological Sciences, University of Calgary, Calgary, Alberta, Canada; University of Maryland, UNITED STATES

## Abstract

*Pseudomonas aeruginosa* is an opportunistic pathogen that often infects individuals with the genetic disease cystic fibrosis, and contributes to airway blockage and loss of lung function. Natural killer (NK) cells are cytotoxic, granular lymphocytes that are part of the innate immune system. NK cell secretory granules contain the cytolytic proteins granulysin, perforin and granzymes. In addition to their cytotoxic effects on cancer and virally infected cells, NK cells have been shown to play a role in an innate defense against microbes, including bacteria. However, it is not known if NK cells kill extracellular *P*. *aeruginosa* or how bacterial killing might occur at the molecular level. Here we show that NK cells directly kill extracellular *P*. *aeruginosa* using NK effector molecules. Live cell imaging of a co-culture of YT cells, a human NK cell line, and GFP-expressing *P*. *aeruginosa* in the presence of the viability dye propidium iodide demonstrated that YT cell killing of *P*. *aeruginosa* is contact-dependent. CRISPR knockout of granulysin or perforin in YT cells had no significant effect on YT cell killing of *P*. *aeruginosa*. Pre-treatment of YT and NK cells with the serine protease inhibitor 3,4-dichloroisocoumarin (DCI) to inhibit all granzymes, resulted in an inhibition of killing. Although singular CRISPR knockout of granzyme B or H had no effect, knockout of both in YT cells completely abrogated killing of *P*. *aeruginosa* in comparison to wild type YT cell controls. Nitrocefin assays suggest that the bacterial membrane is damaged. Inhibition of killing by antioxidants suggest that ROS are required for the bactericidal mode-of-action. Taken together, these results identify that NK cells kill *P*. *aeruginosa* through a membrane damaging, contact-dependent process that requires granzyme induced ROS production, and moreover, that granzyme B and H are redundant in this killing process.

## Author summary

Natural Killer (NK) cells comprise at least 10% of the resident lymphocytes in the lung and are increasingly recognized as an important part of the immune response to bacterial pathogens. Despite *invivo* studies demonstrating the importance of NK cells in the host response to the respiratory pathogen *Pseudomonas aeruginosa*, the mechanism of antimicrobial activity has yet to be found. Using human NK cell lines and NK cells isolated from human peripheral blood, we show that NK cells exhibit direct, contact-dependent cytotoxicity against *P*. *aeruginosa*, leading to bacterial cell death. NK cells use granzyme B and H to damage bacterial membranes and permeabilize the cells. We provide evidence that this leads to increased reactive oxygen species (ROS) in the bacteria that kills them. Furthermore, granzyme function appears to be redundant because loss of function by one granzyme is rescued by the activity of the other. These findings identify a role for granzymes in the antibacterial functions of NK cells, providing new insight into the host response to *P*. *aeruginosa* infections.

## Introduction

*Pseudomonas aeruginosa* is a Gram-negative, facultative anaerobic, opportunistic pathogen that commonly infects individuals with a compromised immune system. *P*. *aeruginosa* infections frequently occur in the respiratory tract, soft tissues, the eyes and ears [1]. The 30 day mortality in nosocomial *P*. *aeruginosa* infections has been reported to be as high as 39% [2] and the mortality from ventilator-associated pneumonia is up to 68% [3]. *P*. *aeruginosa* also affects 40–50% of persons with cystic fibrosis (CF) and acquisition of this pathogen is associated with more rapid deterioration of lung function and risk of death [4]. Consequently, there is great interest in the mechanisms of host defense to *P*. *aeruginosa*.

NK cells are innate lymphoid granulocytes that make up 10–15% of all peripheral blood lymphocytes. NK cells are cytotoxic to malignant or virally infected cells, releasing lytic granules carrying effector molecules that bring about apoptosis in the target cell [5,6]. In addition to their well-described role in tumour and virus infected cell cytotoxicity, NK cells are important in respiratory infections. In the lung, NK cells make up 10% of the resident lymphocytes and many more are recruited within hours (h) after the induction of inflammation [7]. Within the lung, NK cells reside in the interstitium and there they have comparable activity to those in peripheral blood [8]. The alveolar space and airway lumen of a healthy lung are relatively devoid of NK cells [9]. However, after airway infection with bacteria such as *Staphylococcus*, there is a rapid influx of activated NK cells into the airway [9] and functional NK cells can be isolated from human sputa and bronchoalveolar lavage fluid [10,11]. Thus, NK cells play an important role in the host defense against bacterial pathogens of the lung. However, the mechanism by which they exert this activity is unknown.

There is also evidence that murine NK cells are important in *P*. *aeruginosa* infection. Murine NK cells are recovered from bronchoalveolar lavage after *P*. *aeruginosa* infection of the lung [12,13]. Murine models have shown that depletion of NK cells leads to increased mortality as well as an increased burden of *P*. *aeruginosa* in experimental pneumonia [14]. These results clearly support the importance of NK cells in cellular host defense against *P*. *aeruginosa*, but there is limited information on the mechanism of that activity. Determining the specific mechanism of NK cell antimicrobial host defence could inform the development of therapies that for clearance of *P*. *aeruginosa* without the associated tissue damage generated by other immune cell types.

NK cells contribute to host defense in several ways, both indirectly and directly. NK cells produce cytokines such as interferon γ and tumour necrosis factor α, which activate other cells, such as macrophages and neutrophils to kill bacteria [15]. However, this effect is indirect, as these cytokines do not directly kill the organisms. NK cells are generally considered non-phagocytic. A possible exception is NK cells phagocytose and internalize *Candida albicans*; however, in this report killing was extracellular [16]. Conversely, intracellular invasion of NK cells by *P*. *aeruginosa* kills the NK cell via caspase 9-mediated apoptosis [17]. Finally, NK cells have a well-described cytolytic role in the killing of intracellular pathogens. NK cells are activated by ligands present on host cells infected with the bacterium *Listeria monocytogenes* or the protozoans *Trypanosoma cruzi*, *Toxoplasma gondii* and *Leishmania major* to release their cytotoxic granules [18,19]. Additionally, killing of extracellular fungal pathogens by NK cells has been well documented. NK cells recognize and kill the fungal respiratory pathogens *Cryptococcus neoformans*, *Aspergillus fumigatus* and *C*. *albicans* using the cytolytic protein perforin to induce fungal cell death [20–24]. In addition to fungal pathogens, NK cells can kill extracellular *Mycobacterium kansasii* and *Mycobacterium tuberculosis* [25,26]. NK cells have also been previously shown to bind to and directly kill the bacterium *Burkholderia cenocepacia* through a lytic mechanism [27]. Although these results show that NK cells can directly kill extracellular pathogens, it is not known if NK cells kill *P*. *aeruginosa*.

NK cell killing of tumour targets is well defined. Activation of NK cells for tumour cytotoxicity requires a contact-mediated event that leads to the initiation of cellular pathways and the reorganization of secretory granules to be released towards and kill tumour cells. The secretory granules contain the pore forming proteins granulysin and perforin as well as the family of serine proteases, granzymes (Grzms). When killing tumour cells, NK cells use perforin to create pores or damage the target cell membrane allowing Grzms to pass into the target cell [28–30]. The pores formed by perforin allow passive efflux and influx of ions, which disrupt the homeostasis of the target cell [29]. However, the primary pathway of target cell death is induced by Grzms, which activate the caspase pathway and induce mitochondrial reactive oxygen species (ROS) production, resulting in apoptosis [18]. While the roles of each of these effector molecules in tumour killing is relatively well understood, their roles in microbial killing is still a growing area of study.

NK cells utilize their effector molecules to kill intracellular *L*. *monocytogenes*, *T*. *cruzi*, *T*. *gondii* and *L*. *major* [18,31]. Upon release, the effector molecules granulysin and Grzms enter the host cell via perforin-formed pores in the host membrane [18]. Granulysin forms pores in the membrane of pathogens to allow entry of the Grzms [18]. Once in the pathogen, Grzms induce death of the microbe. Studies using recombinant protein directly applied to microbes have reinforced the importance of these effector molecules in target cell death. High concentrations of granulysin are cytolytic to *C*. *neoformans*, *Escherichia coli* and *M*. *tuberculosis*, inducing permeability of the bacterial membrane, which results in the accumulation of fluid in the cytosol causing perturbation of the membrane, leading to cell death [32,33]. Perforin and granulysin have also been shown to kill *Mycobacteria* individually, but an additive effect was seen when both recombinant proteins were added to the culture [25]. In summary, the NK cell cytolytic effector molecules perforin, granulysin and Grzms have each been shown to decrease bacterial viability during intracellular killing [18,19,25,31]. However, it is not known how these effectors work for direct extracellular killing of bacteria such as *P*. *aeruginosa*.

In this study, we investigate the antimicrobial activity of NK cells against extracellular *P*. *aeruginosa*. In order to understand the fundamental mechanisms of NK cell anti-pseudomonal activity, we co-cultured NK cells isolated from the blood of human donors or a human NK cell line (YT) with different strains of *P*. *aeruginosa* in-vitro. Viable cell counts and live cell imaging of NK cells and *P*. *aeruginosa* was used to determine whether there was direct, contact-dependent cytotoxic activity against *P*. *aeruginosa*. We examined the ability to damage the bacterial membrane using nitrocephin conversion and studied the role of granule effector proteins using pharmacologic inhibitors and CRISPR Cas9-induced knockouts. Redundancy of effector proteins was examined using double knockouts, and the association of effector proteins with *P*. *aeruginosa* was studied by isolating the bacteria after exposure to NK cells and performing western blot. Finally, the role of reactive oxygen species was examined using a scavenger of free hydroxyl radicals.

## Materials and methods

### Ethics statement

Experimental protocol for use of human blood were approved and conducted under the guidelines of the Conjoint Health Research Ethics Board of the University of Calgary (certificate number REB13-0057). Written consent was obtained from all participants.

### Human cells

Blood was acquired from healthy volunteers by venous puncture after obtaining consent. Peripheral blood mononuclear cells (PBMCs) were purified by centrifugation on a Ficoll-Hypaque density gradient (GE Healthcare, Mississauga, ON, Canada). Primary human NK (pNK) cells were isolated from PBMCs by negative selection using a manual CD56 MAC’s cell seperation and human CD56 MicroBeads (Miltenyi Biotec, Bergish Gladbach, Germany). Flow cytometric analysis showed more than 95% NK cell purity (CD3^-^CD16^+^CD56^+^). The YT cell line was a gift from Dr. C. Clayberger (National Cancer Institute, Bethesda, MD, USA). All cell lines and primary cells were maintained in RPMI-1640 medium (Thermofisher Scientific, Grand Island, NY) supplemented with 10% heat-inactivated fetal calf serum (Invitrogen Life Technologies, Burlington, Canada), 1% sodium pyruvate (NaPyr) (Invitrogen), and 1% nonessential amino acids (NEAA) (Invitrogen) at 37°C with 5% CO_2_. YT-Lifeact-RFP cells were created by transducing YT cells with a lentiviral pCMVLifeAct-TagRFP virus (Ibidi, Martinsried, Germany). 721.221 cells were obtained from ATCC (Manassas, VA, USA). Prior to experiments with *P*. *aeruginosa*, media was replaced with serum free, RPMI-1640 medium.

### Bacterial strains and growth conditions

All *P*. *aeruginosa* strains and isolates [34] are listed in Table 1. *P*. *aeruginosa* was grown in lysogeny broth (LB; Difco, Le Pont De Claix, France; 24g/L) at 37°C with gentle shaking (220 rpm) overnight prior to each experiment. The overnight culture was then sub-cultured for 4 h at 37°C with gentle shaking prior to the assay. Alternatively, semi-solid plate medium was used for routine strain propagation and was prepared by adding 1.5% w/v Bacto agar to LB. Where appropriate, gentamicin (Gm) was added at 30 μg/ml. to the LB media to select for miniTn7 mutants (Table 1).

**Table 1:** *Pseudomonas aeruginosa* strains and isolates used in this study.

**Strain**	**Genotype, description or relevant characteristics** *	**Source**
JJH0	PAO1 wild type strain originating from the laboratory of Colin Mannoil (MPAO1), genome re-sequenced	[59]
BPTA103	JJH0 *attTn7*::miniTn7T2.1-Gm-GW::*P*_A1/04/03_::GFPmut3*, Gm^r^	[60]
S35004	Blood isolate; Seattle, WA, USA	[34]
CF5	Cystic fibrosis isolate; Columbia, MO, USA	[34]
CF127	Cystic fibrosis isolate; Denver, CO, USA	[34]

*Gm^r^, gentamicin resistance

### Antibodies

For western blot imaging, mouse anti-granulysin (Santa Cruz, Dallas, TX; clone F-9), mouse anti-granzyme B (Biolegend, San Diego, CA), mouse anti human granzyme H clone: 4G5, a generous gift from Dr. Joseph Trapani (Peter MacCallum Cancer Centre, Melbourne, Australia), mouse anti human perforin clone: δG9, secondary antibodies goat anti-mouse IgG infrared dye 800 (Licor, Lincoln, NE), and goat anti-rabbit IgG infrared dye 700DX (Rockland, Limerick, PA) were used for imaging. For flow cytometry experiments, cells were labeled with Alexa Fluor 488 mouse anti-granulysin, clone: RB1 (BD Pharmigen, San Diego, CA), mouse anti granzyme A, clone cb9d (Biolegend), FITC mouse anti granzyme B, clone GB11 (Biolegend), PE-Cy5 mouse IgG1 isotype control (BD Biosciences), FITC mouse anti-Perforin (BD Biosciences), and FITC mouse IgG2b isotype control (BD Biosciences).

### NK/YT cell anti-pseudomonal activity

*P*. *aeruginosa* (500 cells/well, unless otherwise stated) were co-cultured with or without NK cells (pNK or YT: 2-4x10^5^ cells/well) in serum free RPMI media at effector target (ET) ratios of 0 (bacteria alone control), 200 or 400:1 in 96 well round bottom plates. The bacteria inoculum was verified by plating on LB agar. Samples were incubated at 37°C and 5% CO_2_ for 6 h. Co-cultures were then diluted ten-fold in sterile water to lyse the effector cells then spotted on LB agar, which were incubated at 37°C. After 12 h, the colonies were counted and recorded. Each co-culture and ET ratio condition were performed in quadruplicate and experiments (using different donors on different days) were performed in triplicate to ensure reproducibility.

To inhibit granzyme A, YT and pNK cells were pretreated with 0.25 μM (concentrations above 0.50 μM inhibited bacterial growth) 6-amidino-2-naphthyl p-guanidinobenzoate dimethanesulfonate (Futhan; Tocris Bioscience, Bristol, United Kingdom) for 15 minutes prior and during the 6 h co-culture. To inhibit perforin, YT and pNK cells were pretreated with 20 nM concanamycin A (CMA) or DMSO control for 2 h prior to the assay and washed prior to the assay. The serine protease inhibitor 100 μM 3,4-Dichloroisocoumarin (DCI) (Sigma Aldrich) was used to treat YT or NK cells for 30 minutes prior before being removed by washing prior to the killing assay. The granzyme B inhibitor ZAAD-CMK (EMD Millipore, Darmstadt, Germany), was diluted to 10 mM in dimethyl sulfoxide (DMSO; Sigma) and 50 μM was used to pretreat YT and pNK cells for 30 min and kept in the media over the course of the assay. Following pre-treatment with any inhibitors, cell viability was assessed by trypan blue assay and was >95% viability for all experiments.

In the transwell assay experiments, *P*. *aeruginosa* was diluted in (serum free) RPMI media and was placed in the inserts having 0.1μm membrane pores or in the wells (3000 CFU/well) with or without YT cells (1.2x10^6^ cells/well) in 24 well plates. Cells were incubated at 37°C and 5% CO_2_ for 6 h. After incubation, the contents of the wells and transwell inserts were serially diluted one in ten-fold in sterile water to lyse the effector cells. CFU were detected as previously described above. Each co-culture condition was made in triplicate. The experiment was repeated three times to ensure reproducibility.

### Generating CRISPR knockout YT cell lines

YT cells were transfected with target sgRNA (Table 2), non-targeting sgRNA or were mock transfected (Nucleofector, Amaxa) using the protocol provided by the manufacturer for transfection of Cas9 and sgRNA (Synthego). Transfection was done using Nucelofector solution (Cell Line Nucleofector^TM^ Kit V, Lonza, Switzerland). Following transfection, the cells were cultured in 24 well plates at 37 C, 5% CO2. Cells were screened for expression by flow cytometry. Single cells negative for the target protein were sorted into wells in a 96 well plate for clonal selection and expanded. Doublets were excluded based on forward and side scatter, height and area, using the FACSAriaIII (P18100045) machine and FACSDiva version 8.0.1 software. Knockout of the desired gene in expanded clones was confirmed by flow cytometry and western blot.

**Table 2:** CRISPR sgRNA sequences.

**CRISPR KO**	**Target Sequence**	**Clone**	**Parent Clone**
Grzm B	CCUUCAGGGGAGAUCAUCGG	C8	WT YT
Grzm B	CCUUCAGGGGAGAUCAUCGG	D8	WT YT
Grzm B	CCUUCAGGGGAGAUCAUCGG	E9	WT YT
Grzm H	AACAAAGGCCAUGUAGGGGC	F6	WT YT
Grzm H	AACAAAGGCCAUGUAGGGGC	B9	WT YT
Grzm H	AACAAAGGCCAUGUAGGGGC	F10	WT YT
Granulysin	ACCUCCCCGUCCUACACACC	A9	WT YT
Granulysin	ACCUCCCCGUCCUACACACC	D2	WT YT
Granulysin	ACCUCCCCGUCCUACACACC	E6	WT YT
Perforin	ACAGGGGGACUUGGGCUCU	B3	WT YT
Perforin	ACAGGGGGACUUGGGCUCU	D4	WT YT
Perforin	ACAGGGGGACUUGGGCUCU	F8	WT YT
Grzm B&H	UCCUUCAGGGGAGAUCAUCG	G6	GrzmH KO F6 YT
Grzm B&H	UCCUUCAGGGGAGAUCAUCG	G8	GrzmH KO F6 YT
Grzm B&H	UCCUUCAGGGGAGAUCAUCG	G11	GrzmH KO F6 YT
Grzm B&H	UCCUUCAGGGGAGAUCAUCG	F12	GrzmH KO F6 YT

### Live cell imaging

RFP-labelled YT cells (5×10^6^) and GFP PAO1 (Multiplicity of Infection; MOI of 100:1) were added to a sterile 35 mm μ-Dish (Ibidi, Munich, Germany) in a final volume of 300 μL of phenol red and FBS free RPMI media. Propidium iodide (PI; 0.1 mg/mL; Abcam; Catalog no. ab14083) was added to the medium prior to imaging. Images were captured every 15 seconds using the Quorum Wave FX-X1 spinning disk confocal microscope. Images were processed using FIJI, version 2.0.0-rc-54/1.51g.

### Western blot

YT and pNK cells were lysed in of RIPA lysis buffer (Cell Signaling Technology), and supernatants were added to reducing sample buffer. Proteins were separated in a NuPAGE Novex 4–12% Bis-Tris gel (Thermo Fisher) before being transferred to nitrocellulose membranes (Bio-RAD, CA, United States). Antibody at 1:500 in 3% BSA TBST overnight at 4°C was used to reveal the proteins. Mouse monoclonal anti–human beta actin antibody (Chemicon International, Temecula, CA) was used as control. Proteins were visualized by adding secondary IRDye 680RD goat anti-rabbit antibody (Li-COR Inc, Lincoln, NE) or IRDye 800CW goat anti-mouse antibody (Li-COR Inc.). Images were acquired using the Odyssey Imaging System (Li-COR Inc.).

### Flow cytometry

Cells were fixed with 2% paraformaldehyde in PBS, then permeabilized with 1x Permwash solution (BD Biosciences) diluted in DH_2_O for 30 min at 4°C. Samples were then stained with fluorescent-labelled primary antibody in 100 μL of PBS with 3% BSA for 1 h at 4°C. Samples analyzed using Guava EasyCyte™ flow cytometer (EasyCyte, Guava). Flow cytometry was also used to determine membrane permeabilization of *P*. *aeruginosa*. For this purpose, *P*. *aeruginosa* PAO1 was co-cultured with YT cells at the indicated E:T ratios for 4 h in a 37°C CO_2_ incubator. PI (0.1mg/mL) was added into the cell culture medium at the same time as *P*. *aeruginosa*. PI staining was detected using Guava Easy-Cyte flow cytometer (Cytosoft version 5.3, Guava Technologies, Millipore, Danvers, MA), and results were analyzed by FlowJo software v.10 (Tree Star, Ashland, OR). By adjusting the amp gain on the flow cytometer (amp gain of 8 was used for YT cell, and amp gain of 32 for bacteria), bacterial cells were discriminated from the larger YT cells based on size.

### Tumour cell killing

YT cell cytotoxic function against 721.221 cell tumour targets was done using flow cytometry. 721.221 cells were stained with CFSE to distinguish them from YT cells during analysis. YT and 721.221 cells were co-cultured for 4 hrs at 7.5 and 15:1 ET ratios in phenol red and serum free RPMI 1640 media. PI (0.1 mg/mL) was added to the media 5 minutes prior to analysis to stain for dead cells. Samples were analyzed using Guava EasyCyte flow cytometer (EasyCyte, Guava) and FlowJo software v.10.

### Phagocytosis of *P*. *aeruginosa* PAO1

Human donor PBMCs (3x10^6^ cells/well) were cultured for 1 hr in a 24 well plate with RPMI 1640 supplemented with 10% FBS and GM-CSF (40ng/mL; R&D Systems, Minneapolis, MN) in order to differentiate attached cells into macrophage [35]. pNK cells were isolated from the same donor (as previously described) the day prior to the experiment. YT and pNK cells were seeded into the 24 well plate at 3x10^5^ cells/well. Prior to co-culture, all cells were washed with PBS, then media was replaced with serum free RPMI 1640. PAO1 was washed then stained with pHrodo Green AM dye (Thermo Fisher Scientific) as per the manufacturer’s instructions, in PBS on ice for 1hr. The PAO1 was washed then added to the wells at a MOI of 200:1 for 1 hr. Macrophage, pNK and YT cells were then washed with PBS, and samples were run on the flow cytometer to measure green fluorescence, with fluorescence greater than unstained PAO1 control as an indicator of phagocytosis. Samples analyzed using Guava EasyCyte flow cytometer (EasyCyte, Guava) and FlowJo software v.10.

### Nitrocefin experiments

Following a 6 h coculture of *P*. *aeruginosa* and YT or NK cells, 20 μL of nitrocefin at 1mM was added to the wells [36]. The plates were then incubated at 37°C and 5% CO_2_ for 12 hrs. Conversion of nitrocefin was measured at 490 nm using (Spectramax M2).

### Separation of bacteria from co-culture using centrifugation

pNK or YT cells were prepared as above. A 24 well plate was seeded at 1×10^6^ cells/well with and without 1×10^5^ CFU/well of *P*. *aeruginosa* PAO1, followed by a 6 h incubation at 37°C and 5% CO_2_. Following incubation, the wells were washed, and the cells were harvested into 1.5mL tubes. The tubes were then centrifuged at 300 rcf for 5 minutes to sediment the pNK/YT cells. The media containing bacteria was added to a different 1.5mL tube, the supernatant was centrifuged at 2350 rcf for 10 minutes in order to sediment the bacteria in the 10:1 ET ratio co-culture conditions. Lysed pNK/YT cells, bacteria and the supernatants were mixed with 4x NuPAGE LDS Sample Buffer (Novex) Samples were run on western blot gel as previously described. The anti-human beta actin antibody also was used as a control to detect excess pNK/YT cell contamination in the supernatant/ bacterial lysate conditions. The antibody to *E*.*coli* RNA polymerase α (Clone: 4RA2; Biolegend) was added 1:5000 in 3% BSA as a loading control for PAO1.

### Conjugation of YT cells and PAO1

YT cells were labeled with a PE-Cy^TM^ 5 anti-CD11a antibody. YT cells were cocultured with different ratios of GFP-expressing *P*. *aeruginosa* PA01 for 20 minutes in a 37°C, 5% CO_2_ incubator. Conjugates were detected by Guava Easy-Cyte flow cytometer (Cytosoft version 5.3, Guava Technologies, Millipore, Danvers, MA), and results were analyzed by FlowJo software v.10 (Tree Star, Ashland, OR). YT cells were identified by gating on CD11a^+^ cells. The percentage of YT cells conjugated to GFP-expressing *P*. *aeruginosa* strains was determined as the number of events that are PE-Cy5 and GFP positive/number of events PE-Cy5 positive x 100%.

### Statistical analysis

Statistical analysis was performed using GraphPad Prism (GraphPad Software Inc. CA. USA). One-way ANOVA with Bonferroni correction was used to evaluate statistical significance.

## Results

### Primary human NK cells exhibit antimicrobial activity against wild type *P*. *aeruginosa*

**Fig 1:**
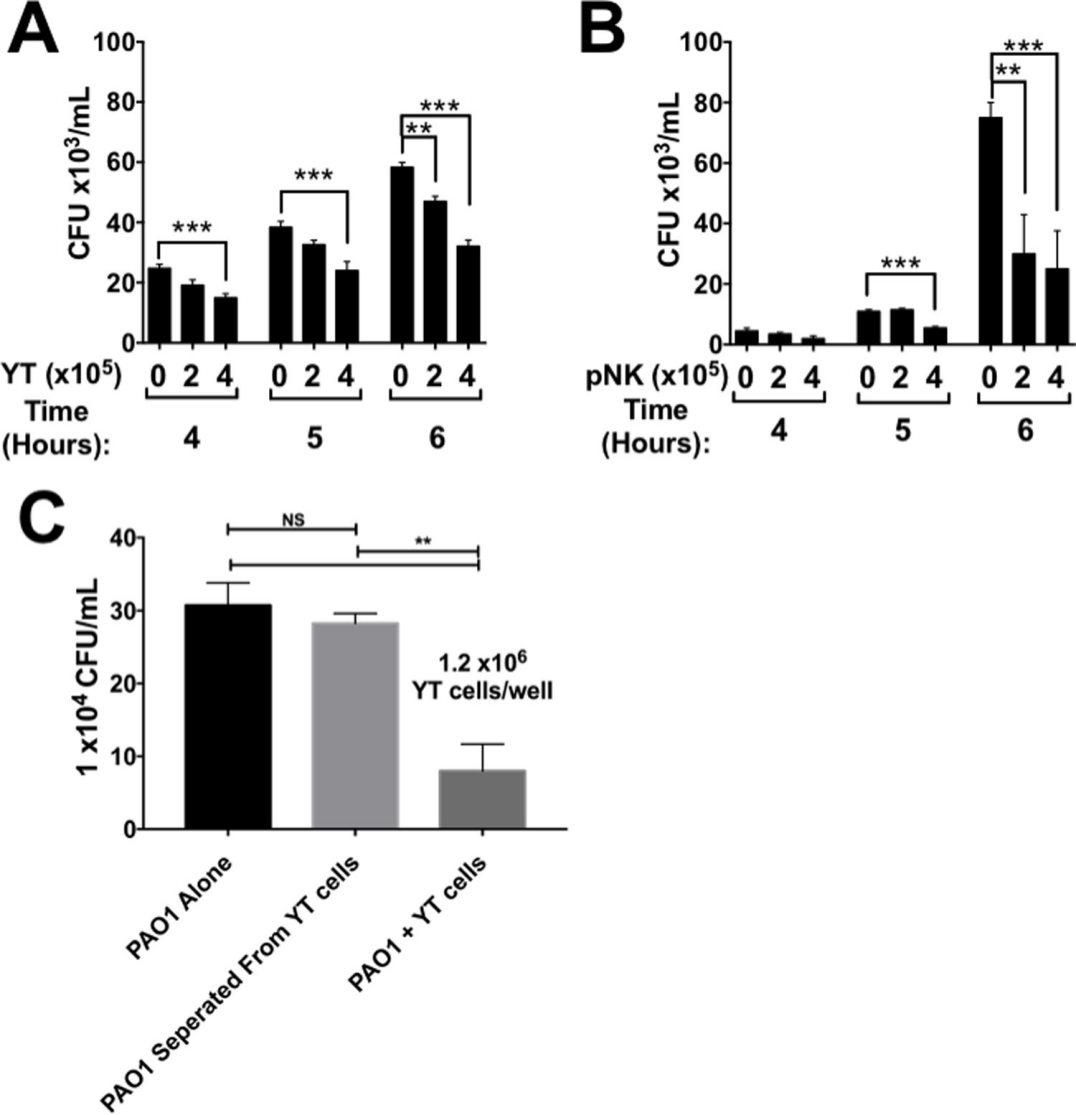
pNK and YT cells have a contact-dependent antimicrobial effect on *P*. *aeruginosa* PAO1. *P*. *aeruginosa* PAO1 cultured alone or in the presence of YT cells (A) or pNK cells (B). (C) CFU of *P*. *aeruginosa* PAO1 incubated alone, separated by a 0.1um filter, or in direct contact with YT cells for 6 h. In all co-culture experiments, conditions were carried out in n = 4 wells (mean ± SEM) and the graph is representative of n ≥ 3 biological replicates performed on different days. ** = P≤0.01, *** = P≤0.001. NS not significant.

To determine if NK cells have direct anti-microbial activity against *P*. *aeruginosa*, we co-cultured the wild-type strain PAO1 with YT and primary NK (pNK) cells at two cell concentrations at three different times (4, 5 and 6 h) (Fig 1A and 1B). The number of PAO1 CFU was reduced significantly in the presence of YT and pNK cells compared to the PAO1 alone (Fig 1A and 1B), increasing numbers of YT or pNK cells causing proportionate reduction in PAO1. When CFU was examined over time, PAO1 growth was significantly reduced in the presence of YT and pNK cells after 5 h, but occurred optimally at 6 h, so this time was chosen for future experiments. This result indicates that both YT cells and pNK cells have an anti-microbial effect on *P*. *aeruginosa*.

We tested if the antimicrobial activity was contact dependent using a transwell assay system with inserts. The pores of the insert allow soluble molecules to freely diffuse through the membrane but will not allow PAO1 to translocate. Using this system, PAO1 was cultured with or without YT cells within the same compartment or separated from YT cells by the insert. PAO1 CFU in the same compartment as YT cells was significantly lower (73.98% ± 23.90) than PAO1 separated from YT cells or alone in culture (Fig 1C). To address whether soluble effector molecules released due to bacteria-stimulated degranulation of YT cells, but not in contact with PAO1 can kill the bacteria, we use a modified transwell assay (S4 Fig). In the experiment, a co-culture of PAO1 and YT cells in a well was separated from PAO1 in the transwell insert. We found that CFU of the PAO1 in contact with YT cells were reduced, while CFU in the transwell insert that were not in contact were not reduced (S4 Fig). These results reveal that the anti-microbial effect is contact dependent and is not due to constitutive release of a soluble compound by YT cells that inhibits bacterial growth.

**Fig 2:**
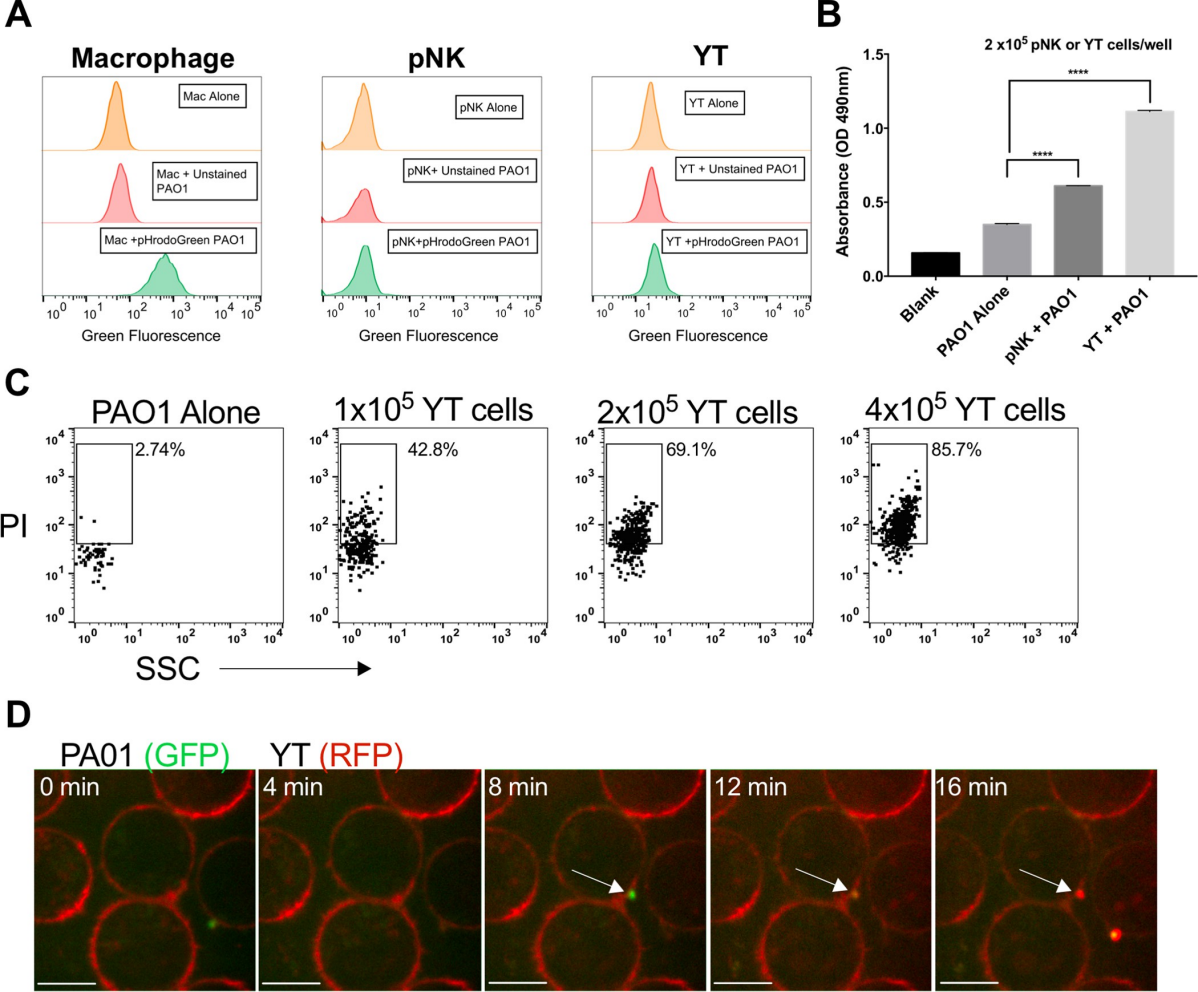
pNK and YT cells do not phagocytose *P*. *aeruginosa* PAO1, but they do directly induce membrane damage. (A) Human macrophage, pNK or YT cells co-cultured with or without PAO1 at a 200:1 MOI for 1 hr. PAO1 was either unstained or stained with pHrodo Green AM dye to measure phagocytosis in the different cell types. A shift in green fluorescence as measured by the flow cytometer indicates phagocytosis of PAO1 by the cell type. Graph is representative of N = 2 biological replicates carried out on different days. Gating strategy in S4 Fig. (B) PAO1 incubated alone or in the presence of YT or human primary NK cells for 6 h followed by addition of 1mM nitrocefin for 12 h. (C) Representative flow cytometry plot of Propidium iodide (PI) staining of PAO1 that were either cultured alone or with YT cells for 2 h in culture media containing PI. (D) YT cells, expressing RFP actin, co-cultured with GFP PAO1. Membrane permeability was determined by the addition of PI to the media. Images were acquired every 15 seconds and representative images are shown at 2-minute intervals. Scale bar = 10μm. The video (Supplementary Video 1) is representative of at least 3 separate experiments. In all co-culture experiments, conditions were carried out in n = 4 wells (mean ± SEM) and the graph is representative of n ≥ 3 biological replicates performed on different days. * = P≤0.05, ** = P≤0.01, *** = P≤0.001. NS not significant.

To quantify bacterial conjugation to NK cells in culture, we labeled YT cells with red fluorescent CD11a antibody and co-cultured with GFP-expressing wild type PA01 and determined by flow cytometry the percentage of YT cells with bound bacteria (fluoresced red and green). We found that with increasing numbers of bacteria, fluorescent intensity increased revealing that increasing numbers of *P*. *aeruginosa* became bound to each YT cell (S5A Fig). Previous studies had shown that NK cells phagocytose *C*. *albicans* after a 30 minute incubation [16], so we tested if phagocytosis was the method of bactericidal activity against *P*. *aeruginosa*. pNK, YT and human macrophage cells were exposed to pHrodo Green AM stained PAO1. pHrodo Green AM stained PAO1 are non-fluorescent when at a neutral pH, but when exposed to acidic environments such as that in a phagolysosome, it fluoresces green. We co-cultured the PAO1 for 1 h with human macrophage that were differentiated from PBMCs as a positive control for phagocytosis. We observed that following the co-culture, the majority of the macrophage population exhibited green fluorescence, indicating phagocytosis and phagolysomal processing of the bacteria, but no green fluorescence was detected in pNK and YT cells (Fig 2A). This suggested phagocytosis and intracellular killing was not the mechanism of anti-pseudomonal activity.

NK cell antimicrobial activity has conventionally been achieved when its cytolytic effector molecules permeabilize the membrane of its target [16,18,23]. To determine whether the outer membrane (OM) of PAO1 was damaged following co-culture with pNK and YT cells, we used a colorimetric assay based on the processing of the cephalosporin, nitrocefin, by beta lactamases within the periplasm of Gram-negative bacteria (Fig 2B). The OM is normally impermeable to nitrocefin, but when it is damaged, nitrocefin gains access to the periplasm where it is converted from yellow to red, with color change to red indicating outer membrane permeability [37,38]. The absorbance readings showed that there was a significant increase in the conversion of nitrocefin when pNK and YT cell were co-cultured with bacteria compared to PAO1 alone (Fig 2B). Nitrocefin conversion was increased by 75.1% ± 2.28 in the presence of pNK compared to PAO1 alone, while YT cells caused a 219% ± 4.64 increase (Fig 2B). This demonstrates that pNK and YT cells induce significant damage to the outer membrane of the PAO1.

To assess the damage to the bacterial inner membrane, we assessed membrane permeabilization using propidium iodide (PI) as a marker of bacterial death. PI is a membrane impermeant dye that stains DNA when both the outer and inner bacterial membranes become permeable during cell death. We cultured wild-type PAO1 with YT cells at a series of effector to target (ET) ratios and added PI to the culture medium. Using flow cytometry, we found that 43% of the PAO1 population when cultured with YT cells (Fig 1E). The percentage of PI^+^ PAO1, and thereby bacterial cell death, increased in a dose dependent manner with increasing numbers of YT cells (Fig 2C). We also observed YT cells directly causing membrane damage to *P*. *aeruginosa* by live-cell imaging. YT cells engineered to express the red fluorescent protein (RFP) on actin structures were cultured with GFP-expressing *P*. *aeruginosa* PAO1 and immediately imaged (Fig 2D and S1 Video). Only those PAO1 cells that bind to YT cells become PI+ and the membrane failure indicating bacterial cell death took 5–10 minutes. Additionally, PAO1 membrane damage happened when one YT cell contacted one bacterium (ET ratio of 1:1) (Fig 2D and S1 Video). This experiment also serves to determine the number of YT cells in contact with each PAO1. While experiments assessing the ET ratio raised the possibility that multiple YT cells might be required to engage each PAO1 cell, the live cell imaging clearly shows that only one YT cell engages one PAO1 cell to kill it. In summary, NK and YT cells killed *P*. *aeruginosa* PAO1 in a direct, contact dependent process resulting in membrane permeability and bacterial death.

### NK cell killing of clinical isolates

**Fig 3:**
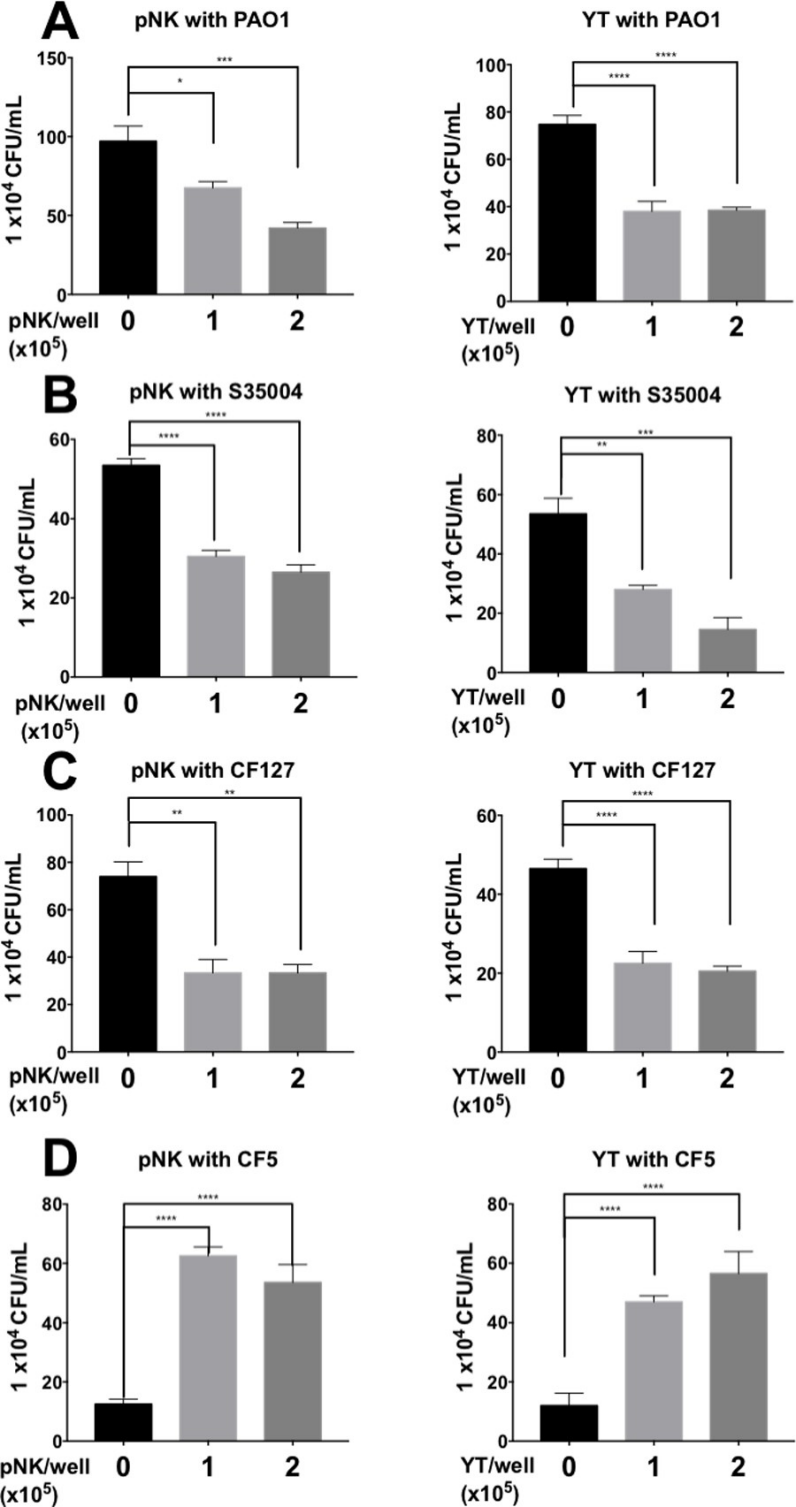
pNK and YT cells kill some clinical isolates of *P*. *aeruginosa* in addition to PAO1. Co-culture of pNK or YT cells with strain PAO1 (A), strain S35004, a blood isolate (B) or CF strains CF127 and CF5 (C & D). CFU was determined by plate counting. Conditions were carried out in n = 4 wells (mean ± SEM) and the graph is representative of n ≥ 3 biological replicates performed on different days. * = P≤0.05, ** = P≤0.01, *** = P≤0.001. NS not significant.

NK and YT cells demonstrated antimicrobial activity against the *P*. *aeruginosa* lab strain PAO1. We sought to determine if the pNK and YT cells also killed clinical isolates of *P*. *aeruginosa*. pNK and YT cells were cultured with two CF isolates (CF5 and CF127) and one sepsis isolate (S35004). Both NK and YT cells killed CF127 and S35004, demonstrating similar, cell concentration-dependent killing as PAO1 (Fig 3A, 3B and 3C). However, the CF5 isolate demonstrated resistance to both NK and YT cell killing and grew significantly more in co-culture than in the bacteria alone control condition (Fig 3D). We wondered if CF5 was killing YT cells, but trypan blue viability of YT cells following 6 h co-culture revealed no difference (S1 Fig). While NK and YT cells can kill multiple clinical isolates, the inhibition of killing of CF5 revealed that anti-pseudomonal activity is strain dependent.

### Perforin and granulysin are not involved in NK and YT cell killing of *P*. *aeruginosa*

**Fig 4:**
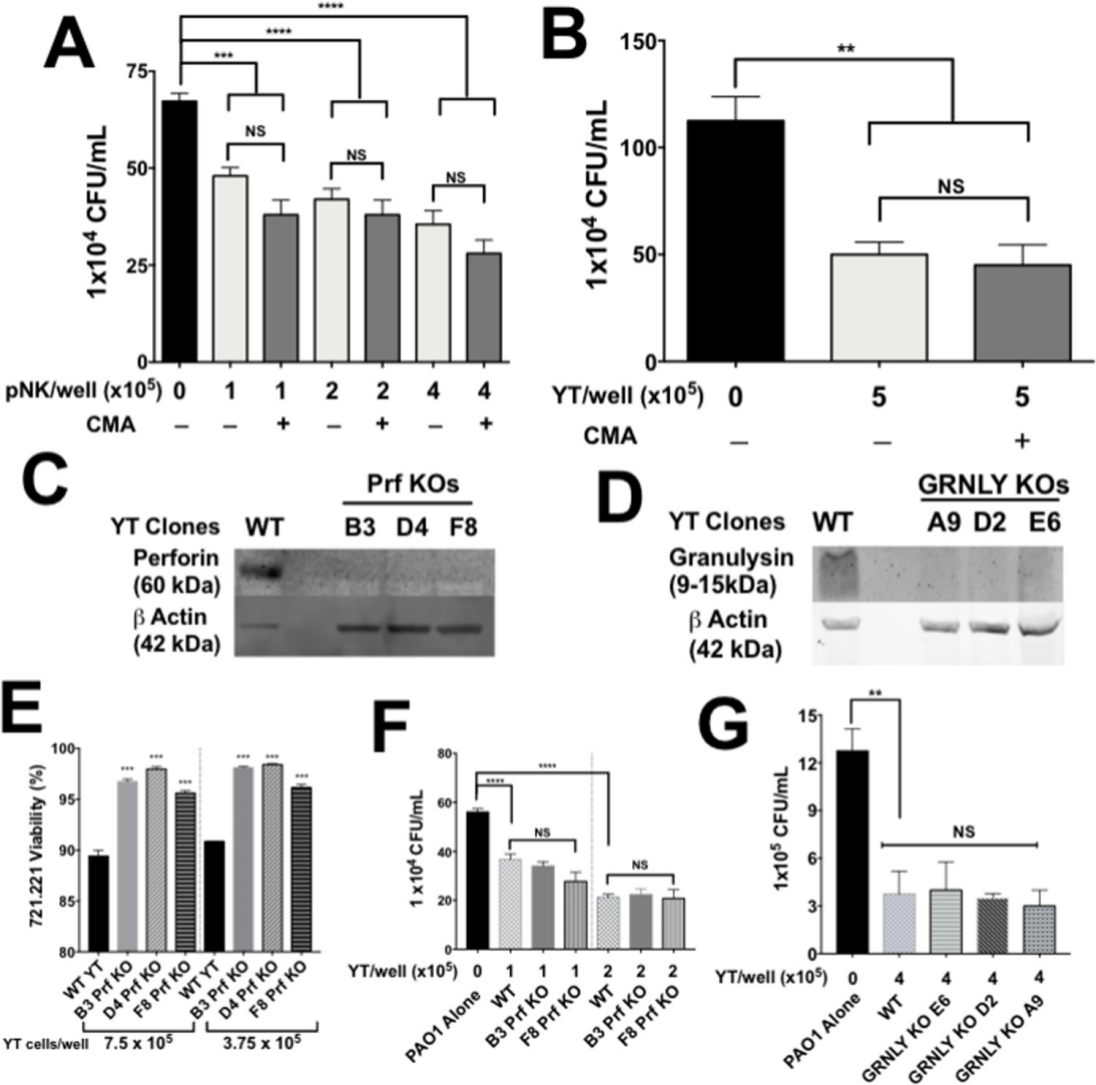
Perforin and granulysin are not required in YT cell killing of PAO1. pNK (A) and YT (B) cells were treated with concanamycin A (CMA) or control for 2 h to inhibit the perforin-mediated cytotoxic pathway. PAO1 (1000 CFU/well) was added for 6 h. CFU counts were used to determine number of viable PAO1. Western blot of wild type or Prf KO YT cells (5x10^5^ cells) (C) or wild type or GRNL KO YT cells (5x10^5^ cells) (D) were blotted using (1:1000) mouse antibodies specific for human perforin (Clone: δG9) or human granulysin (clone: F-9). Rabbit antibody specific for human beta actin was used as a loading control. (E) YT cell cytotoxicity against 721.221 tumour target cells. 722.221 cells were stained with CFSE prior to the assay to distinguish them from effector cells. The membrane permeability dye 7AAD was used to assess tumour cell death. Percent killing was determined by the percentage of red and green fluorescent cells divided by the total green fluorescent population. Conditions were carried out in n = 3 wells (mean ± SEM) and the graph is representative of n ≥ 2 biological replicates. (F) PAO1 incubated alone or in the presence of wild type or perforin knockout YT cells and cultured for 6 h, then plated for CFU counts. (G) PAO1 (1000 CFU/well) incubated alone or in the presence of wild type or granulysin knockout YT cells and cultured for 6 h, then plated to determine CFU. In all co-culture experiments, conditions were carried out in n = 4 wells (mean ± SEM) and the graph is representative of n ≥ 3 biological replicates. * = P≤0.05, ** = P≤0.01, *** = P≤0.001.

The previous experiments suggested that killing was due to a direct cytotoxic process involving permeabilization of the bacterial inner and outer membranes. This led us to examine the role of NK cell effectors that increase membrane permeability in mammalian cytotoxicity. The first effector molecule we tested was perforin. Perforin is required for the direct killing of a several bacterial pathogens by NK cells [25,31]. Concanamycin A (CMA), which blocks processing of perforin to its active form by inhibiting the acidification of the secretory granules, was used to inhibit the perforin-mediated cytotoxic pathway. Previous work showed that incubation of YT and NK cells with 20nM CMA for 2 h successfully inhibits the perforin cytotoxic pathway [23]. YT and pNK cells were pretreated with CMA prior to the co-culture. YT and pNK cell viability as determined by trypan blue staining was greater than 95% in both the treated and untreated conditions. *P*. *aeruginosa* killing by CMA-treated YT and pNK was not significantly different from cells treated with DMSO (control) (Fig 4A and 4B). Since CMA-treated YT and pNK cells were able to kill PAO1, it suggests that NK cell killing of PAO1 is independent of the perforin cytotoxic pathway.

To further verify the results of the CMA experiments, we used CRISPR to knockout perforin in YT cells. Three clones (B3, D4 and F8) of perforin CRISPR knockout (Prf KO) YT cells were generated (Fig 4C). Three clones were used in subsequent experiments to help eliminate possible misleading off target effects. All 3 Prf KO clones had significant inhibition in their cytotoxicity against 721.221 tumour targets (Fig 4E). The significant inhibition of YT cell cytotoxicity against tumour targets demonstrates that the knockout has an impaired cytotoxic function due to the loss of perforin. However, the Prf KO YT clones killed PAO1 to a similar extent as the WT YT parent line (Fig 4F), showing YT cell killing of PAO1 does not require perforin.

Like perforin, granulysin causes cell death by damaging the membrane of the microbial cell and cellular injury [39]. Granulysin has previously been shown to be an important effector molecule in the direct killing of extracellular fungi [25,33]. Granulysin has also been shown to permeabilize *P*. *aeruginosa* membranes, leading to bacterial cell death [40]. Three granulysin CRISPR knockout clones (granulysin KO) were generated to test if granulysin was required for YT cell killing of PAO1 (Fig 4D). All 3 granulysin KO clones killed PAO1 as effectively as the WT YT parent line (Fig 4G). Taken together, these findings show that YT cell killing of PAO1 does not require the pore forming proteins perforin or granulysin.

### Granzymes are required for NK cell killing of *P*. *aeruginosa*

**Fig 5:**
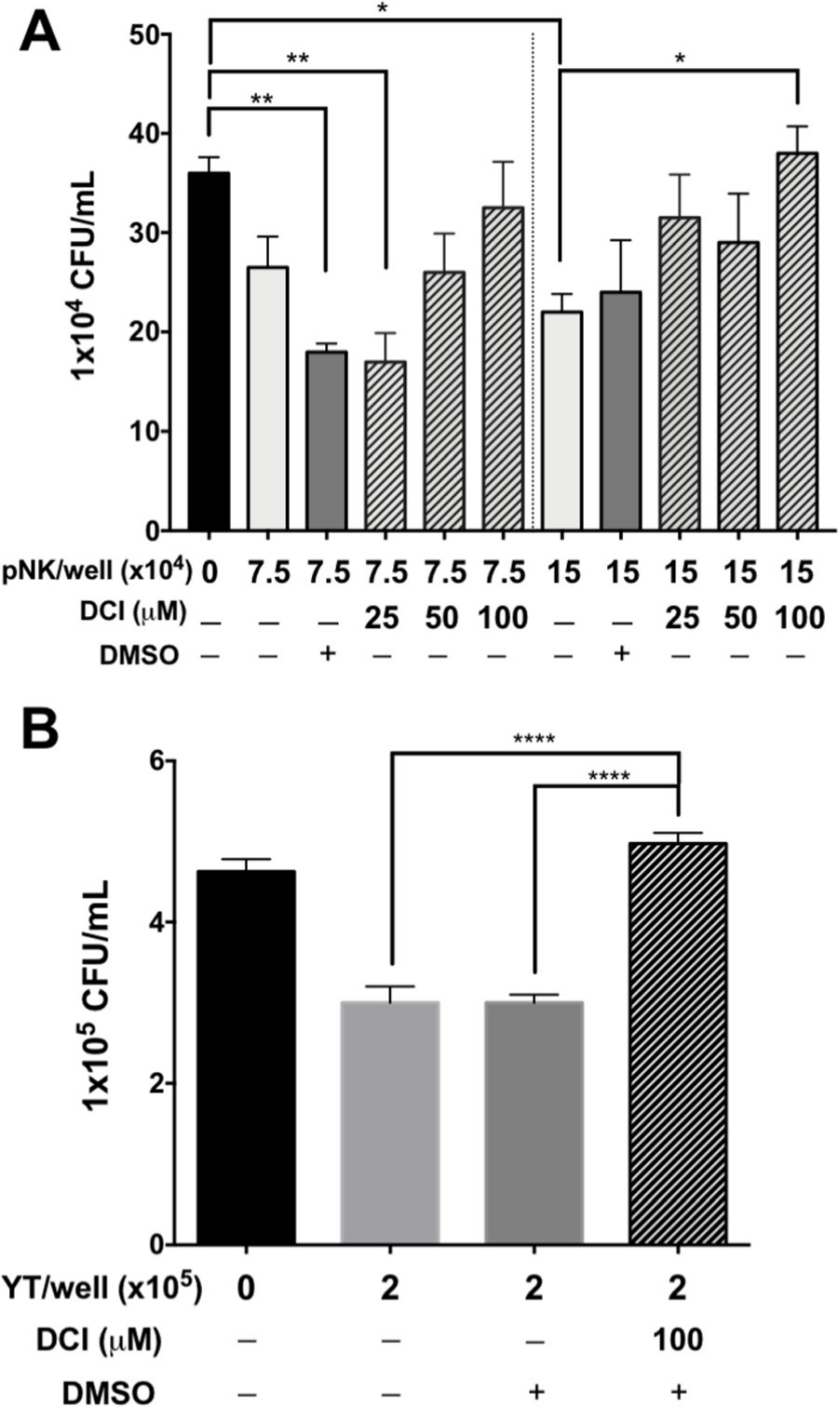
Inhibition of serine proteases abrogates killing in pNK and YT cells. PAO1(1000 CFU/well) incubated alone or in the presence of pNK (A) or YT cells (B) for 6 h before determining CFU. pNK or YT cells were pre-treated with 3,4-Dichloroisocoumarin (DCI) for 1 h to inhibit the serine proteases, including granzymes. Conditions were carried out in n = 4 wells (mean ± SEM) and the graph is representative of n ≥ 3 biological replicates * = P≤0.05, ** = P≤0.01, *** = P≤0.001.

Granzymes (Grzms) are serine proteases and major effectors of NK cells. To determine whether Grzms play a role in the direct killing of PAO1 by NK cells, we used the serine protease inhibitor DCI (3,4-dichloroisocoumarin) to irreversibly inhibit Grzm activity [41]. YT and NK cells were pretreated with DCI or an equivalent volume of DMSO vehicle and monitored for their ability to kill PAO1 in a standard CFU killing assay. YT and pNK cell killing of PAO1 was inhibited by DCI in a dose dependent manner (Fig 5A and 5B). This was not the result of DCI toxicity as YT and pNK cell viability were not significantly changed following treatment with DCI at any dose as determined by trypan blue staining. These results indicate that serine proteases, which include, Grzms are involved in NK cell killing of *P*. *aeruginosa*.

### YT and pNK cells express granzyme B and H

**Fig 6:**
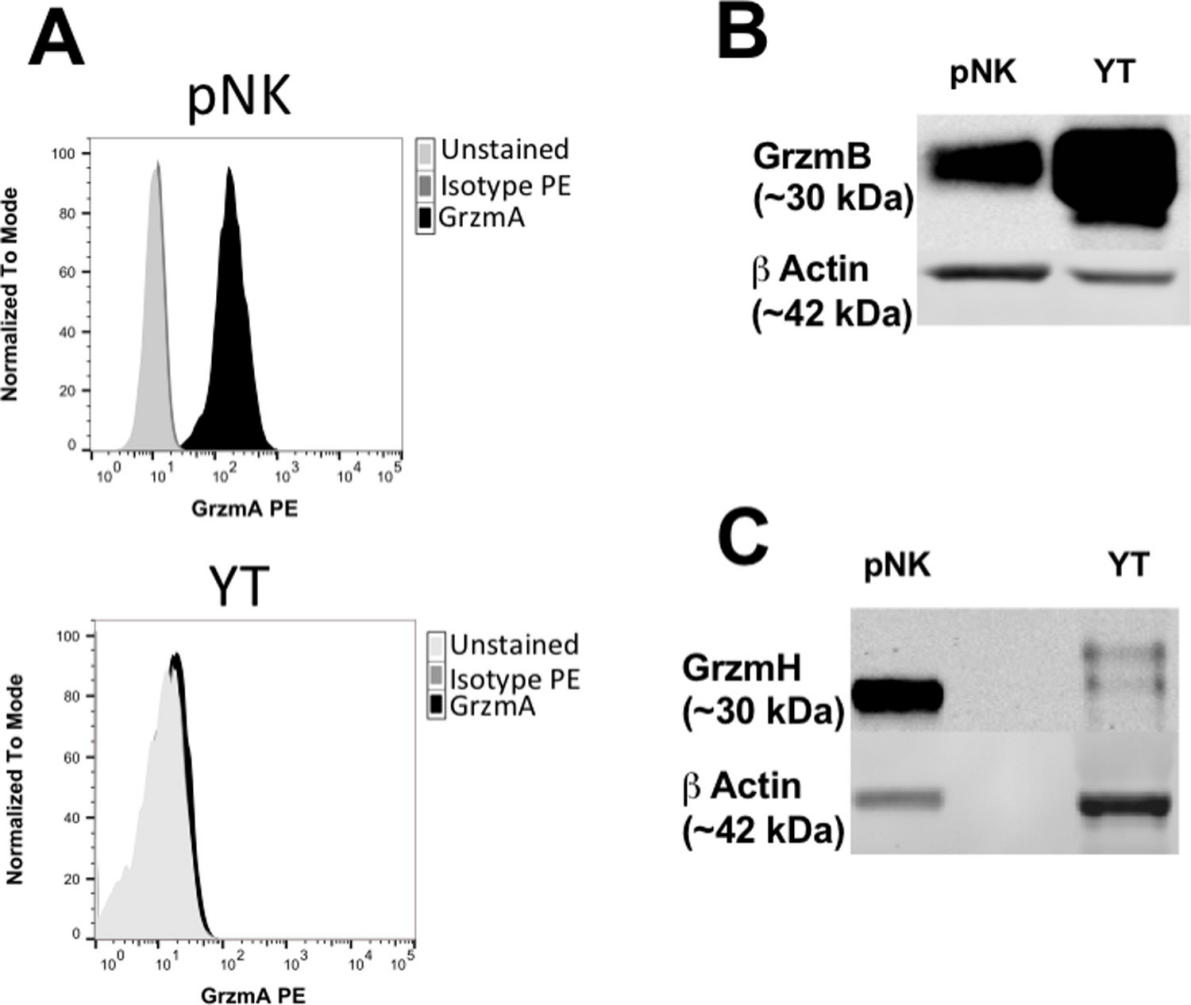
pNK and YT cells both express GrzmB and H. (A) Human pNK or YT cells were fixed with 2% PFA, permeabilized, then stained with mouse antibody (1:200) specific for Grzm A followed by a rabbit anti-mouse antibody conjugated to FITC (green histogram). Unstained YT and pNK cells (blue histogram) labelled with isotope IgG1 primary antibody and rabbit anti-mouse antibody conjugated to FITC (red histogram) were used as negative controls. pNK (1x10^6^ cells) or wild type YT cells (5x10^5^ cells) blotted using (1:1000) mouse antibodies specific for human Grzm B (B) or H (C). Rabbit antibody specific for human beta actin antibody was used as a loading control.

Grzms are differentially expressed in lymphocyte subsets and their respective cell lines. To determine what Grzms are expressed by YT and NK cells, flow cytometric and/or western blot analysis were conducted on pNK and YT cells depending upon the validated application of the available antibodies. In our studies we used an antibody for Grzm A for flow cytometric analysis (Fig 6A) and antibodies specific for Grzm B and H were used for western blot analysis (Fig 6B and 6C). Flow cytometric analysis showed that Grzm A was expressed by pNK cells and not detected in YT cells (Fig 6A). These results were also consistent with the literature [42]. Western blot analysis of pNK lysates showed similarly high levels of Grzm H and Grzm B expression (Fig 6C). This finding was consistent with some, but not all studies in human NK cells [42–44]. Western blot analysis of YT cell lysates showed high levels of Grzm B expression, but lower Grzm H expression (Fig 6B and 6C). These results were used to direct our subsequent experiments. We hypothesized that Grzm B was the likely candidate responsible for killing as it was present in both pNK and YT cells and had previously been shown to have bactericidal activity, whereas Grzm H has not been demonstrated to have a role in bactericidal activity [18,19,45].

### Multiple granzymes are required for NK antimicrobial activity

**Fig 7:**
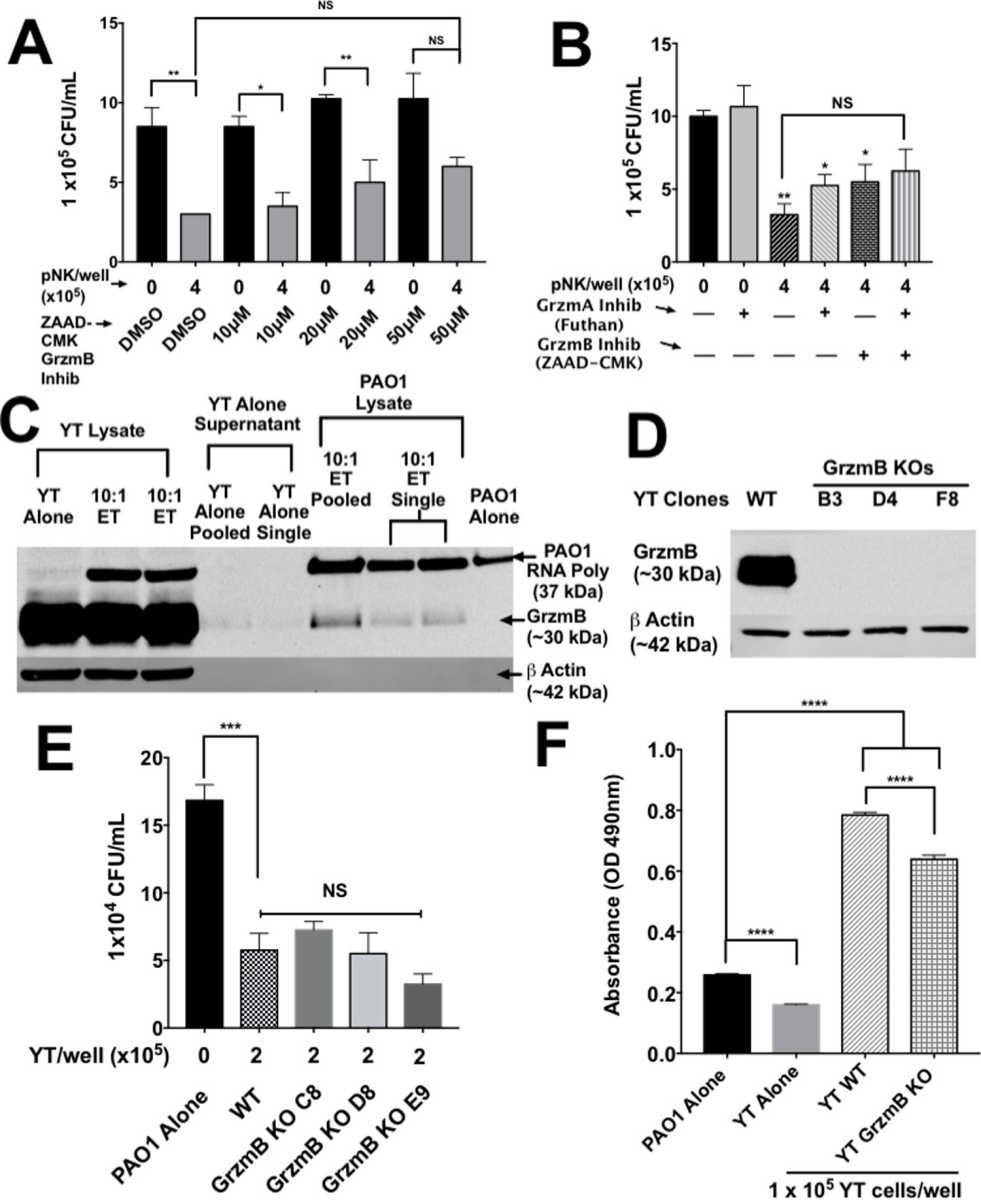
Single KO of GrzmB does not affect YT cell killing of PAO1. (A) PAO1 (1000 CFU/well) incubated alone or in the presence of pNK cells treated with various concentrations of the granzyme B inhibitor ZAAD-CMK or DMSO (control). (B) Untreated pNK treated with 50μM ZAAD-CMK, 0.25μM granzyme A inhibitor (Futhan) or both and cultured for 6 h then plated to determine CFU. * = P≤0.05 and ** = P≤0.01. (C) Western blot analysis of Grnz B in YT cells (1x10^6^ cells), PAO1 or culture media from 6 h co-cultures at a 10:1 ET ratio. PAO1 was separated from YT cells and culture media using a series of centrifugation and washes with sterile distilled water to separate the bacteria which were lysed with 2x Sample Buffer and boiled. Bands were revealed using (1:1000) mouse antibodies specific for human Grzm B. Rabbit antibody specific for human beta actin and a rabbit antibody specific was *E*. *coli* RNA polymerase were used as loading controls for the YT and PAO1 cells respectively. (D) Western blot analysis of wild type and granzyme B knockout (C8, D8 and E9) YT cells (5x10^5^ cells). Blotted using (1:1000) mouse antibodies specific for human Granzyme B (clone: m3304b06). (E) PAO1 incubated alone or in the presence of wild type or GrzmB knockout YT cells and cultured for 6 h before determining CFU. (F) PAO1 incubated alone or in the presence of WT or GrzmB KO YT cells for 6 h. Nitrocefin (1mM) was added for 12 h. In all co-culture experiments, conditions were carried out in n = 4 wells (mean ± SEM) and the graph is representative of n ≥ 3 biological replicates. * = P≤0.05, ** = P≤0.01, *** = P≤0.001.

Grzm B is well characterized and is expressed in YT and pNK cells. To determine whether Grzm B played a role in killing, we tested YT and pNK cell killing of PAO1 in the presence of the Grzm B inhibitor ZAAD-CMK. ZAAD-CMK is an irreversible, cell permeable, specific inhibitor of Grzm B [46]. Inhibition of Grzm B using ZAAD-CMK failed to inhibit killing of PAO1 at higher doses (Fig 7A). However, we were unable to exclude Grzm B, because while ZAAD-CMK is highly specific, it is not as strong of an inhibitor as DCI [46].

Grzm A is expressed in pNK cells and has demonstrated bactericidal effects [18]. We tested the involvement of Grzm A in pNK cell killing of PAO1 by targeted inhibition. The Grzm A inhibitor futhan, was added to pNK cells in an attempt to inhibit killing of PAO1 [47]. Killing of PAO1 by pNK cells was not inhibited by futhan, or futhan and ZAAD-CMK together (Fig 7B). Since we failed to detect Grzm A expression in YT cells and failed to reduce killing by inhibiting Grzm A in pNK cells, we concluded that Grzm A was not involved in killing of PAO1.

Since DCI inhibited killing, we asked whether Grzm B was taken up by the bacteria during the killing process. To determine whether Grzm B is released and associates with *P*. *aeruginosa*, low speed centrifugation was used to remove the YT cells, followed by high-speed centrifugation to separate the bacteria from the supernatant. Western blot of the bacterial pellet revealed Grzm B in PAO1 that had been incubated with YT cells compared to PAO1 alone (Fig 7C). This showed that Grzm B was associated with the bacteria (either on the cell surface or internalized) following co-culture. This association justified generation of Grzm B knockout YT cell clones. Three Grzm B CRISPR knockout clones (GrzmB KO) were generated to test if GrzmB was required for YT cell killing of PAO1 (Fig 7D). The PAO1 cell counts when co-cultured with all 3 GrzmB KO clones were similar to those when co-cultured with WT YT and significantly lower than the bacteria alone control (Fig 7E). This confirms the ZAAD-CMK result and reveals that YT cell killing of PAO1 does not require GrzmB or that it is functionally redundant with another granzyme. However, the ability of GrzmB KO YT cells to cause OM damage was reduced compared to WT cells, suggesting that GrzmB played a role in OM damage despite its inhibition or knockout having no effect on killing (Fig 7F). These findings reveal that GrzmB is either taken up by or binds to PAO1 and that it plays a role in outer membrane permeability of PAO1, but knockout does not abrogate killing.

**Fig 8:**
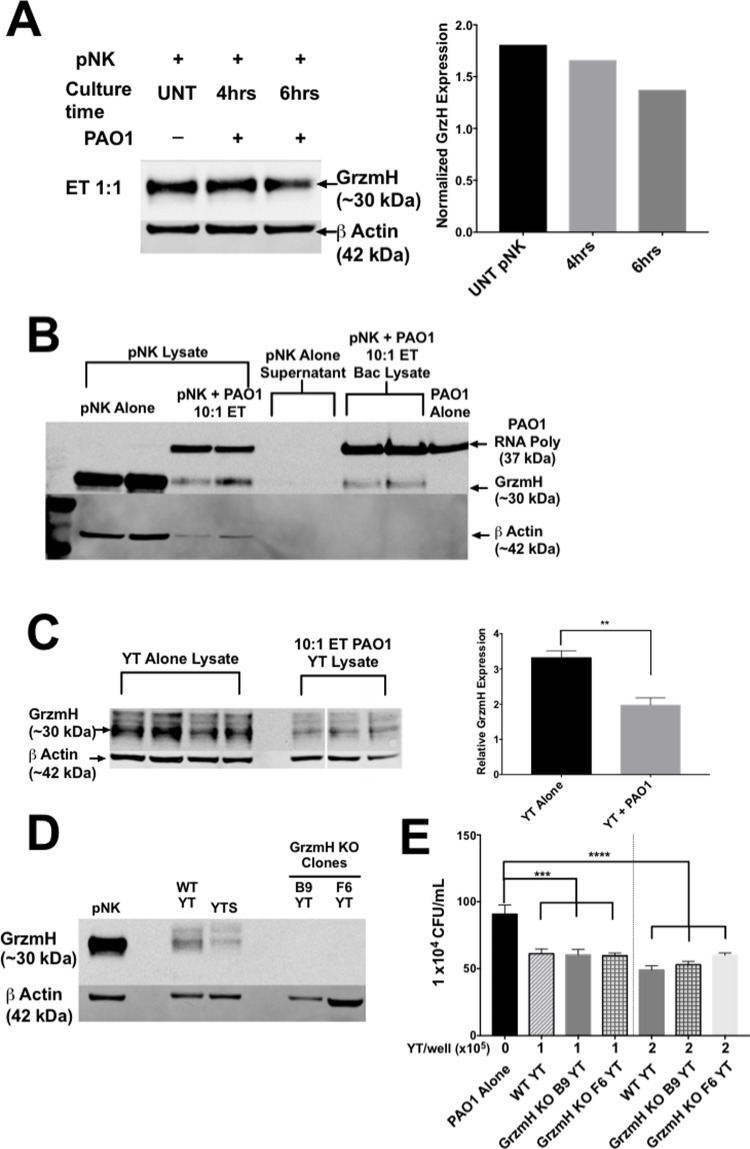
Single KO of GrzmH does not affect YT cell killing of PAO1. (A) Western blot and densitometry of pNK cells (1x10^6^ cells), alone or co-cultured with 1x10^6^ cells bacteria for either 4 or 6 h. Blotted using (1:1000) mouse antibodies specific for human GrzmH. Rabbit antibody specific for human beta actin antibody was used as a loading control. Intracellular GrzmH levels were normalized to beta actin for densitometry. (B) Western blot analysis of pNK cells (1x10^6^ cells), alone or co-cultured with 1x10^5^ PAO1 for 6 h. PAO1 was isolated from the co-culture using a series of centrifugation and washes with sterile distilled water to form a bacterial pellet which was lysed with 2x Sample Buffer and then boiled. Blotted using (1:1000) mouse antibodies specific for human GrzmH. Rabbit antibody specific for human beta actin and a rabbit antibody specific was *E*. *coli* RNA polymerase were used as loading controls for the pNK and PAO1 cells respectively. (C) Western blot and densitometry of YT cells (5x10^5^ cells), alone or cultured with 4x10^5^ PAO1 for 6 h. Blotted using (1:1000) mouse antibodies specific for human Grzm H. Rabbit antibody specific for human beta actin antibody was used as a loading control. Intracellular GrzmH levels were normalized to beta actin for densitometry. (D) Western blot analysis of wild type or GrzmH KO YT cells (human leukemia NK cell line) (5x10^5^ cells) and blotted using (1:1000) mouse monoclonal antibodies specific for human GrzmH (4G5). Rabbit antibody specific for human beta actin antibody was used as a loading control. (E) PAO1 incubated alone or in the presence of wild type or GrzmH knockout YT cells and cultured for 6 h, then plated for CFU counts. Conditions were carried out in n = 4 wells (mean ± SEM) and the graph is representative of n ≥ 3 biological replicates * = P≤0.05, ** = P≤0.01, *** = P≤0.001.

The results so far indicated that GrzmB is important in outer membrane damage, but not for killing, suggesting the involvement of additional effector molecules. We examined the role of Grzm H because it is highly expressed in the cytoplasmic granules of pNK cells and to a lesser extent in YT cells. Grzms B and H are both located at the same loci on chromosome 14 and have very similar amino acid sequences and three-dimensional structure [42]. However, Grzm H is a chymase that cleaves after aromatic residues and is thought to induce apoptosis in mammalian cells by inducing mitochondrial ROS production [43]. pNK cells, which contain GrzmH in cytoplasmic granules, show a loss of intracellular Grzm H following a 6 h co-culture with PAO1 at a 1:1 ET ratio consistent with degranulation (Fig 8A). Furthermore, following centrifugation to separate the bacteria from pNK cells and supernatants, western blot analysis of the bacterial pellet demonstrated a presence of Grzm H with the bacteria (Fig 8B). This result suggested that Grzm H becomes associated with or internalized by the bacteria following co-culture. YT cells were also shown to have significant loss of intracellular Grzm H following a 6-h co-culture with PAO1 consistent with degranulation (Fig 8C). These results suggested that Grzm H may be playing a role in anti-pseudomonal activity of NK cells. However, Grzm H is not as well characterized as other Grzms and there are no commercially available specific inhibitors. To test its role in YT cell killing of PAO1, we generated Grzm H CRISPR knockout clones (GrzmH KO) (Fig 8D). In co-culture experiments, PAO1 cell counts in the Grzm H KO WT YT condition were similar to those in the WT YT condition and significantly lower than the PAO1 alone control (Fig 8E). This result shows that the GrzmH is taken up by PAO1 but the knockout of GrzmH does not result in a loss of killing of PAO1 by YT cells.

**Fig 9:**
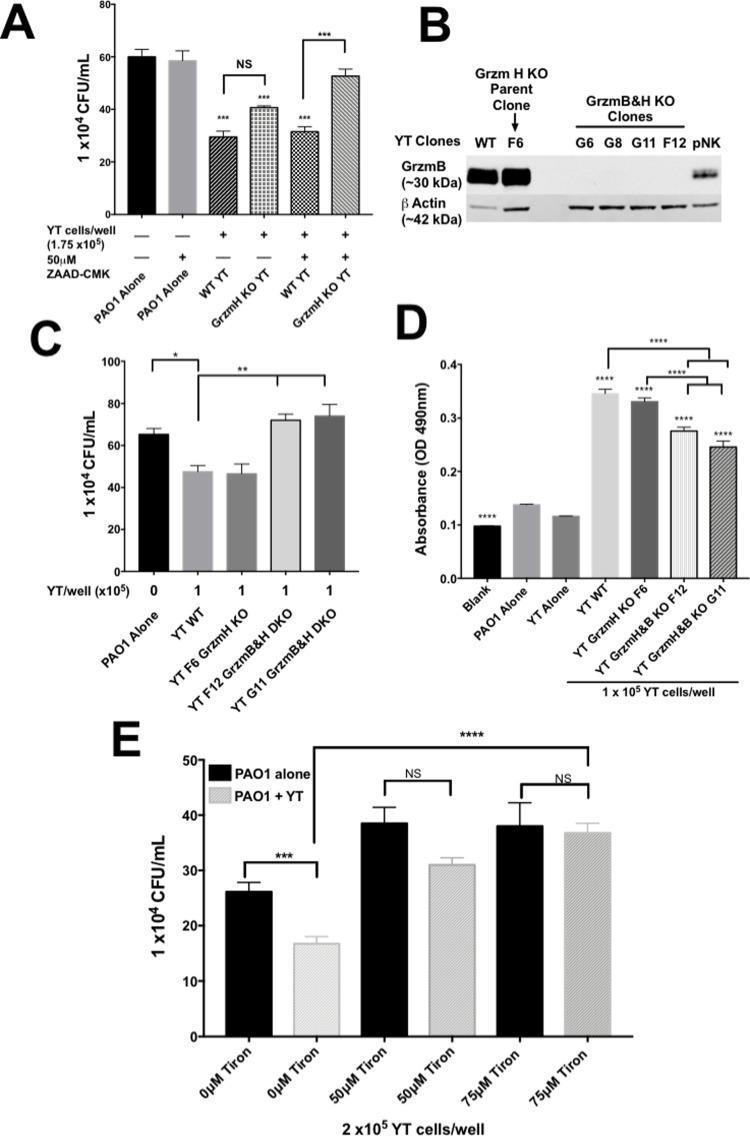
YT cells kill PAO1 using GrzmB&H. (A) PAO1 incubated alone or in the presence of wild type or GrzmH knockout YT cells, treated with 50μM ZAAD-CMK or DMSO (vehicle control) and cultured for 6 h, then plated to determine CFU. (B) Western blot analysis of WT, F6 GrzmH KO, GrzmB&H KO YT (5x10^5^ cells) and pNK cells (1.4x10^6^ cells). Blotted using (1:1000) mouse antibodies specific for human GrzmB (clone: m3304b06) and rabbit antibody specific for human beta actin antibody was used as a loading control. (C) PAO1 incubated alone or in the presence of wild type, GrzmH KO or GrzmB&H knockout YT cell clones YT cells for 6 h then plated to determine CFU. Graph is representative of 2 of the 4 clones tested. All clones demonstrated similar killing ability. (D) PAO1 incubated alone or in the presence of WT, GrzmH KO or GrzmB&H KO YT for 6 h, then diluted 1:10 in sterile distilled water and 1mM nitrocefin and incubated for 12 h. (E) PAO1 incubated alone or in the presence of YT cells with or without Tiron, a ROS scavenger, at various concentrations for 6 h the plated to determine CFU. In all co-culture experiments, conditions were carried out in n = 4 wells (mean ± SEM) and the graph is representative of n ≥ 3 biological replicates. * = P≤0.05, ** = P≤0.01, *** = P≤0.001.

The literature reveals that Grzm function can be redundant [43,48,49]. To determine whether GrzmB and GrzmH function redundantly to kill *P*. *aeruginosa*, Grzm H KO cells were treated with ZAAD-CMK to inhibit Grzm B and killing was determined. The addition of a Grzm B inhibitor to Grzm H KO cells abrogated killing compared to YT cells that were treated with ZAAD-CMK or untreated YT cells (Fig 9A), suggesting that inhibition of killing only occurs when multiple Grzms were inhibited. To validate this finding, we generated 4 CRISPR double KO GrzmB&H clones from the F6 GrzmH KO YT cell line (which did not abrogate killing) (Fig 9B). GrzmB&H double KO clones demonstrated significantly less killing of *P*. *aeruginosa* compared to WT or F6 (single Grzm H) cells (Fig 9C). Indeed, GrzmB&H double KO YT cells failed to reduce *P*. *aeruginosa* cell counts compared to bacteria alone revealing that no significant killing occurred (Fig 9C). This finding reveals that knockout of GrzmB&H in YT cells results in a loss of ability to kill PAO1. The fact that inhibition of killing was found in all 4 KO clones (Figs 9C and S2) indicates that the effect was unlikely to be due to an off-target effect. The loss of GrzmB&H did not affect YT cell viability in comparison to WT YT, following the 6-hour co-culture with PAO1, as determined by a trypan blue assay (S6 Fig). Furthermore, GrzmB&H double KO cells had reduced nitrocefin cleavage, indicating that damage to the outer membrane of PAO1 was reduced compared to WT and GrzmH KO YT cells (Fig 9D).

Since Grzms can exert their effect by inducing ROS, we tested the effect of scavenging free hydroxyl radicals on killing. Tiron, a ROS scavenger, was added to the media during NK cell killing of *P*. *aeruginosa* at two different concentrations (50μM and 70μM). Killing was measured by counting CFU. There was a significant inhibition of killing when 50 μM or 70 μM Tiron was used in comparison to the control condition without Tiron (Fig 9E). This result suggests that ROS plays a role in the killing of PAO1. In summary, both Grzm B and H are functionally redundant in NK cell killing, via a process that leads to ROS production.

## Discussion

In this study, we have made a several key observations regarding the mechanisms of NK cell anti-pseudomonal activity. We found that NK cells have direct, contact-dependent cytotoxic activity against *P*. *aeruginosa*. Despite damage to the bacterial outer and plasma membranes of *P*. *aeruginosa*, the pore forming proteins perforin and granulysin were not required for NK cell anti-pseudomonal activity. Multiple granzymes are functionally redundant for NK cell killing of *P*. *aeruginosa*. Loss or inhibition of a single granzyme did not reduce NK cell anti-pseudomonal activity, only loss of both granzymes B and H had an effect. Overall, we found that NK cells kill extracellular *P*. *aeruginosa* through a contact-dependent process involving granzyme release and ROS.

*P*. *aeruginosa* is a major opportunistic and nosocomial pathogen. NK cells play a largely overlooked role in the host defense against *P*. *aeruginosa* infections. NK cell direct killing of extracellular fungal pathogens is well characterized [20,21,24,33,50], but killing of extracellular bacteria is novel. In murine models, depletion of NK cells leads to an increased susceptibility to *P*. *aeruginosa* pneumonia [14]. While NK cell effector molecules, which are contained in cytoplasmic secretory granules traffic to the plasma membrane and are released, resulting in antimicrobial activity against several pathogens as we previously showed using live cell imaging [18,25,32,40,45,51], the mechanism of extracellular direct anti-bacterial activity of NK cells has not been elucidated.

Previous work demonstrates that NK cells can phagocytose *C*. *albicans* as an anti-microbial defence mechanism [16]. NK cells phagocytosed *Candida albicans* within 30 minutes of interaction, but did not affect the viability of *C*. *albicans*. There are no studies showing uptake and killing of any bacteria by NK cells. In the phagocytosis experiments, we used macrophage, pNK and YT cells cultured with PAO1 to demonstrate that no phagocytosis of the bacteria occurred in the NK cells. Instead, we found multiple lines of evidence that the killing of *P*. *aeruginosa* occurred extracellularly. Live cell imaging experiments showed NK cell killing of extracellular bacteria in contact, but not internalized in the NK cell. Additionally, flow cytometric analysis of propidium iodide uptake by *P*. *aeruginosa* was gated on extracellular bacteria, not NK cells that might contain bacteria providing additional evidence that killing was extracellular. There was a loss of intracellular granzymes during the bacterial killing, indicating that the granzymes were released from the NK cells into the extracellular space to kill the bacteria. If the granzymes had been deployed intracellularly, we would have expected no net loss in granzyme content by the NK cells. Finally, we identified granzymes associated with bacteria by western blot. In these experiments, NK cells were not lysed to liberate the bacteria. Instead, extracellular bacteria were separated by centrifugation, indicating that granzymes were deployed in the extracellular space. Together, these experiments indicate that killing was extracellular and that phagocytosis and internalization were not involved in the killing process.

We showed that pNK or YT cells have an antimicrobial effect on *P*. *aeruginosa* PAO1 and other clinical isolates. Our results demonstrated that YT cells directly induce membrane permeability indicating bacterial cell death. Killing occurred within 10–15 minutes of YT cell contact with the bacterial cell and live cell imaging showed a 1:1 interaction without internalization resulting in death of the bacterial cell. We demonstrated that this direct killing involves damage to the bacterial membranes. We were able to exclude killing by secondary metabolites produced by YT cells by using transwell inserts in which YT cells were separated from *P*. *aeruginosa* by a 0.1 μm membrane. Indeed, the number of viable bacterial cells was significantly greater than the *P*. *aeruginosa* that was cultured directly with YT cells. Although it is not clear whether membrane permeability is a primary or secondary event leading to bacterial death, these results show that an individual NK cell contacts a single bacterium and quickly leads to a catastrophic event.

Although most clinical and lab isolates of *P*. *aeruginosa* were susceptible to NK cell killing, the CF isolate CF5 grew significantly more in the presence of both pNK and YT cells. This finding suggests that the CF5 isolate may have evolved a way of evading either the anti-microbial function or avoid detection and activation of the NK cells. More work will need to be done to determine how *P*. *aeruginosa* virulence mechanisms influence NK cell anti-microbial effects.

After demonstrating that perforin and granulysin were not required, we explored the role of other NK cell effector molecules in killing. We investigated the role of Grzms, which are serine proteases, in killing of *P*. *aeruginosa* by NK cells. Addition of purified Grzms to bacteria has previously been shown to have an anti-bacterial effect through a variety of pathways, including the production of bacterial ROS and the inhibition of bacterial metabolism [18,19]. To our surprise, knockout of GrzmB failed to block the killing. However, knockout of GrzmH with inhibition of GrzmB and double KO of GrzmB&H in YT cells significantly inhibited killing of *P*. *aeruginosa*. These results reveal a major role for GrzmH that has not previously been identified and demonstrates that Grzms are required and functionally redundant for NK cell killing of *P*. *aeruginosa*.

The previous paradigm stated that granzyme anti-microbial activity require the presence of a pore forming protein to gain access to the intracellular compartment, which allowed the granzymes to exert their effects on microorganisms [18,19]. During extracellular killing, we failed to demonstrate a role for perforin and granulysin in NK cell killing of *P*. *aeruginosa*, revealing the role pore forming proteins is limited to intracellular bacteria. Moreover, pore forming proteins are not required to damage the bacterial membrane to facilitate Grzm entry into the bacterial cell. We demonstrated that Grzms become associated with (either bound to the surface or internalized) PAO1 following co-culture with NK cells. This raises the possibility that Grzms gain entry into the bacteria through alternative means. We found that damage to the outer membrane requires GrzmB, and although this occurred prior to bacterial cell death, the mechanism of membrane permeability is unclear. The outer membrane of *P*. *aeruginosa* plays an important role in selective permeability and houses the outer membrane proteins responsible efflux of antimicrobial agents. One possibility is that granzymes directly damage the outer membrane through cleavage of proteins embedded in the membrane, resulting in increased outer membrane permeability and allow access to the periplasm. GrzmB KO YT cells induced significantly less outer membrane damage to *P*. *aeruginosa* than WT YT cells. Previous work has shown that a number of secreted and outer membrane-bound bacterial virulence-related proteins in the intracellular pathogens *L*. *monocytogenes*, *M*. *tuberculosis* and *Salmonella typhimurium*, are cleaved by granzyme B [45]. We found that decreased outer membrane permeability, relative to WT YT cells, was not seen in GrzmH KO cells, suggesting the effect was specific to GrzmB. However, GrzmB KO YT cells were able to kill at comparable levels to WT YT cells, which indicates Grzms do not solely rely on outer membrane damage for killing. In the mitochondria (which shares an evolutionary similarity with prokaryotes) Grzms pass through the outer and inner mitochondrial membranes via protein channels [52]. This passive efflux into the periplasm or cytosol would allow for Grzms to access many potential substrates for cleavage. Therefore, it is conceivable that Grzms could function independently and do not require a pore forming protein in order to gain entry to the bacterial cytosol. In summary, Grzms may either passively diffuse through the membranes via outer membrane pores or induce damage themselves in order to gain entry.

Once inside, the Grzms can cleave subunits of the electron transport chain (ETC) and generate ROS [18]. We demonstrated that ROS is necessary for NK cell killing of *Pseudomonas*. ROS have been shown to damage the bacterial proteins, DNA and membranes [18,53,54]. Grzms could be inducing ROS, which could damage the bacterial membrane indirectly. Damage by the ROS could induce additional membrane permeability in the bacteria, which could explain the difference in nitrocefin conversion in WT vs GrzmB&H KO YT cells. Grzms not only induce ROS, but cleave proteins used by the bacterial cell that quench ROS, inhibiting the bacteria’s ability to recover from the ROS damage [18]. GrzmB cleaves the *E*.*coli* bacterial proteins involved in quenching ROS in the cytosol of the cell, further accelerating the damage of ROS [18]. Homologues for these ROS scavenging proteins were found in *P*. *aeruginosa* [55]. Perhaps the damage induced by Grzms is more effective than a membrane damaging molecule like granulysin as its effect on the bacterial cell is multi-faceted, affecting multiple different processes within the cell and impairing its ability to recover from the damage. In summary, both Grzm B&H function redundantly in NK cell killing of *P*. *aeruginosa*, gaining entry into the bacterial cell without the presence of pore forming proteins.

As previously stated, our results in both YT and pNK cells showed that inhibition of killing requires knockout or inhibition of multiple granzymes (specifically GrzmB and GrzmH). These experiments reveal that Grzms B and H have a redundantly significant role in NK cell killing of *P*. *aeruginosa*. Redundancy in this situation is defined by the loss of function of one granzyme, being rescued by a different granzyme in the cell. There is some evidence of granzyme redundancy in the literature. Tumour killing experiments using Grzm KO’s in mice have demonstrated minimal differences between single Grzm KO strains, but complete loss of anti-tumour activity in double GrzmA&B KO, indicating necessity and redundancy in Grzm function [43]. Perhaps the greatest example of Grzm redundancy lies in humans. In humans, a congenital absence or loss of function of one of the Grzms has not been identified. This may be because a single mutation would be compensated by the highly redundant function of the 5 members of the family. It follows that multiple Grzms are used by YT cells to kill *P*. *aeruginosa*, but they exhibit redundancy in their function. Consequently, multiple Grzms must be inhibited or knocked out in order to abrogate *P*. *aeruginosa* killing. The results in YT cells must be extended with caution to pNK cells, which have a slightly different expression profile of Grzms. We showed that pNK cells express marginally different amounts GrzmA, B and H relative to YT cells. Due to the lack of commercial inhibitor for GrzmH we were unable to specifically inhibit its function in pNK cells. However, as we have shown that GrzmH is important in YT cell killing, and pNK cells express even higher levels of it intracellularly, it is likely that GrzmH is important in pNK cell killing. Overall, it appears that both YT and pNK cell killing makes use of multiple Grzms in kill *P*. *aeruginosa*.

We showed that Grzms exhibit a redundant function in the killing of PAO1, but we also know that they exert different effects on the bacterial cell. For example, we showed that GrzmB KO YT cells induced significantly less outer membrane damage to PAO1 than WT YT cells. However, GrzmH KO YT cells induced similar damage to the outer membrane as WT YT cells. While, the granzymes are redundant in their overall function of generating ROS in the bacteria, they exert different effects on the bacterial cell. Particular target proteins of GrzmA and B in Gram-negative bacteria have been identified, but target bacterial proteins of GrzmH are not known [19]. This study is the first to describe an antibacterial function of GrzmH. While the novel finding of the antimicrobial activity of GrzmH enlists interesting avenues of study, the contributions of the five individual granzymes in PAO1 killing is unclear at this time. There is some evidence of Grzms may work synergistically in antiviral activity. In adenovirus infections, GrzmH cleaves adenoviral proteins that inhibit GrzmB activity [56]. With each Grzm having a distinct cleavage residue, there are a wider array of targets in the microorganism that could be cleaved. Even if an organism evolves a mutation to avoid GrzmB cleavage on an essential protein, it is unlikely that the mutation also protects the molecule from cleavage by GrzmH. It is also possible that depending on the organism, certain granzymes may play a larger role. Previous work done in mice showed that different granzymes played a larger role in the host defence against specific pathogens in particular tissues [57,58]. For example, some pathogens could be more susceptible to GrzmB rather than GrzmH depending on the distinct amino acid sequences present in its proteins. This could mean that certain granzymes are more important in infections of one type of bacterial pathogen than they are in an infection from a different pathogen. Although multiple granzymes could carry out redundant functions, one granzyme may be more effective than the others. For example, we showed that GrzmB generates outer membrane damage in addition to ROS generation, whereas GrzmH does not induce outer membrane damage. More work needs to be done to determine what the contribution of individual granzymes have on killing of PAO1 and potentially other bacteria.

In summary, our results demonstrate a novel role for NK cells in the host response against the pathogen *P*. *aeruginosa*. This work extends the previous observations that NK cells kill fungi. It is possible that Grzm-mediated NK cell killing may extend to other pathogens. Moreover, this study demonstrates the functionally redundant requirement for Grzms in killing of extracellular bacteria via production of reactive oxygen species.

## Supporting information

S1 Video YT cells induce direct membrane damage to *P*. *aeruginosa* following binding. YT cells, expressing RFP actin were co-cultured with GFP PAO1. Propidium iodide was added to the media, which caused the bacteria to fluoresce red when membrane integrity was lost and PAO1 was killed. Images were acquired every 15 seconds. Scale bar = 10μm. The video (Supplementary Video 1) is representative of at least 3 separate experiments.
(ZIP)

S1 Fig YT viability is not altered following co-culture with *P*. *aeruginosa*. YT cells co-cultured with either *P*. *aeruginosa* PAO1 or the CF isolate CF5 for 6 h. After co-culture, the YT cell viability was assessed using trypan blue staining. Conditions were carried out in n = 4 wells (mean ± SEM) and the graph is representative of n = 1 biological replicates.
(TIFF)

S2 Fig YT cells kill PAO1 using GrzmB&H. PAO1 incubated alone or in the presence of wild type, GrzmH KO or GrzmB&H knockout YT cell clones (A) G6 or (B) G8 YT cells for 6 h then plated to determine CFU. In all co-culture experiments, conditions were carried out in n = 4 wells (mean ± SEM) and the graph is representative of n ≥ 3 biological replicates. * = P≤0.05, ** = P≤0.01, *** = P≤0.001.
(TIFF)

S3 Fig Gating strategy for phagocytosis of PAO1 *P*.*aeruginosa*. Human macrophage, pNK or YT cells co-cultured with or without PAO1 at a 200:1 MOI for 1 hr. Gating based on forward and side scatter. Graph is representative of N = 2 biological replicates carried out on different days.
(TIFF)

S4 Fig Modified transwell assay. CFU of *P*. *aeruginosa* PAO1 incubated alone, separated by a 0.1um filter, or in direct contact with YT cells for 6 h. In all co-culture experiments, conditions were carried out in n = 3 wells (mean ± SEM) and the graph is representative of 2 biological replicates performed on different days. ** = P≤0.01, *** = P≤0.001. NS not significant. Illustration created by DDF using BioRender.
(TIF)

S5 Fig Increased conjugation of *P*. *aeruginosa* to YT cells with increasing ratios of bacteria to effector cells. YT cells were labeled with PE-Cy5-CD11a and co-cultured with GFP-expressing PA01 for 10 minutes at the indicated ratios. Flow cytometry analysis was performed to determine the percentage of CD11a^+^ YT bound to GFP^+^ *P*. *aeruginosa*. Gating was done using YT cells, and populations that fluoresced positive for both red and green were considered conjugates, and the degree of binding was normalized to isotype control. Representative scatter plot showing the percentage of the three *P*. *aeruginosa* strains bound to YT cells.
(TIF)

S6 Fig WT and GrzmB&H KO YT cell viability is not altered following co-culture with *P*. *aeruginosa*. WT or GrzmB&H KO YT cells co-cultured with *P*. *aeruginosa* PAO1 for 6 h. After co-culture, the YT cell viability was assessed using trypan blue staining. Conditions were carried out in n = 4 wells (mean ± SEM) and the graph is representative of n = 1 biological replicates.
(TIF)
