# Supplementary figures and images for: Natural killer cells kill extracellular Pseudomonas aeruginosa using contact-dependent release of granzymes B and H
Source: PLoS Pathog. 2022 Feb 24;18(2):e1010325. doi: 10.1371/journal.ppat.1010325 (PMC8903247; doi:10.1371/journal.ppat.1010325)

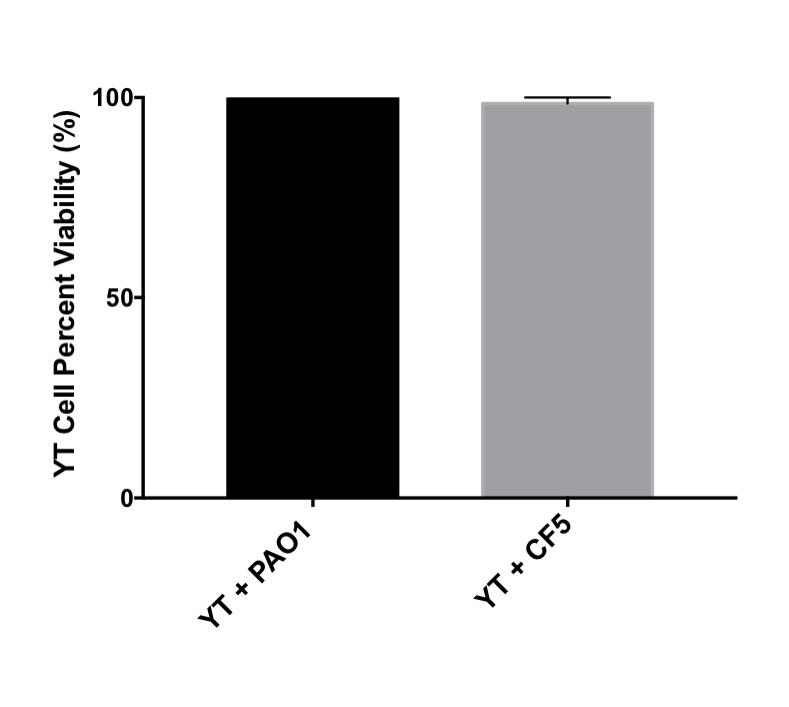

Supplement: S1 Fig — YT cells co-cultured with either P. aeruginosa PAO1 or the CF isolate CF5 for 6 h. After co-culture, the YT cell viability was assessed using trypan blue staining. Conditions were carried out in n = 4 wells (mean ± SEM) and the graph is representative of n = 1 biological replicates. (TIFF) [file ppat.1010325.s002.tiff]

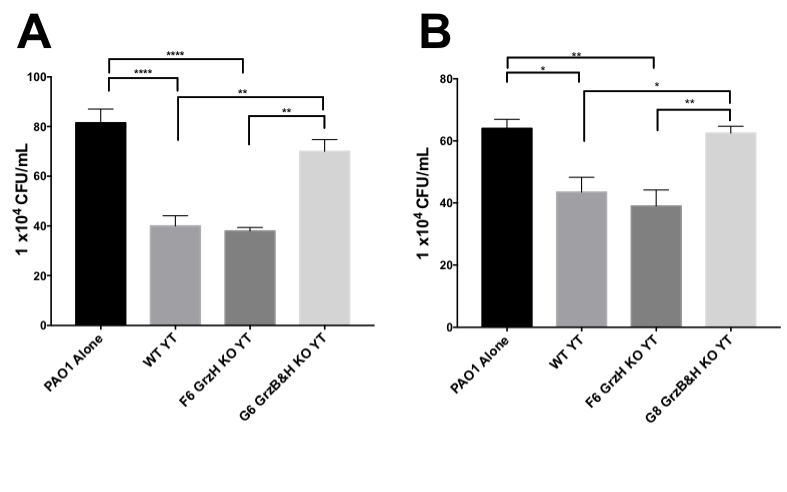

Supplement: S2 Fig — PAO1 incubated alone or in the presence of wild type, GrzmH KO or GrzmB&H knockout YT cell clones (A) G6 or (B) G8 YT cells for 6 h then plated to determine CFU. In all co-culture experiments, conditions were carried out in n = 4 wells (mean ± SEM) and the graph is representative of n ≥ 3 biological replicates. * = P≤0.05, ** = P≤0.01, *** = P≤0.001. (TIFF) [file ppat.1010325.s003.tiff]

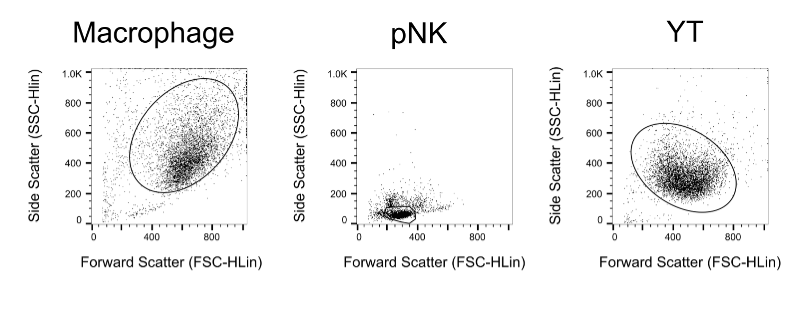

Supplement: S3 Fig — Human macrophage, pNK or YT cells co-cultured with or without PAO1 at a 200:1 MOI for 1 hr. Gating based on forward and side scatter. Graph is representative of N = 2 biological replicates carried out on different days. (TIFF) [file ppat.1010325.s004.tiff]

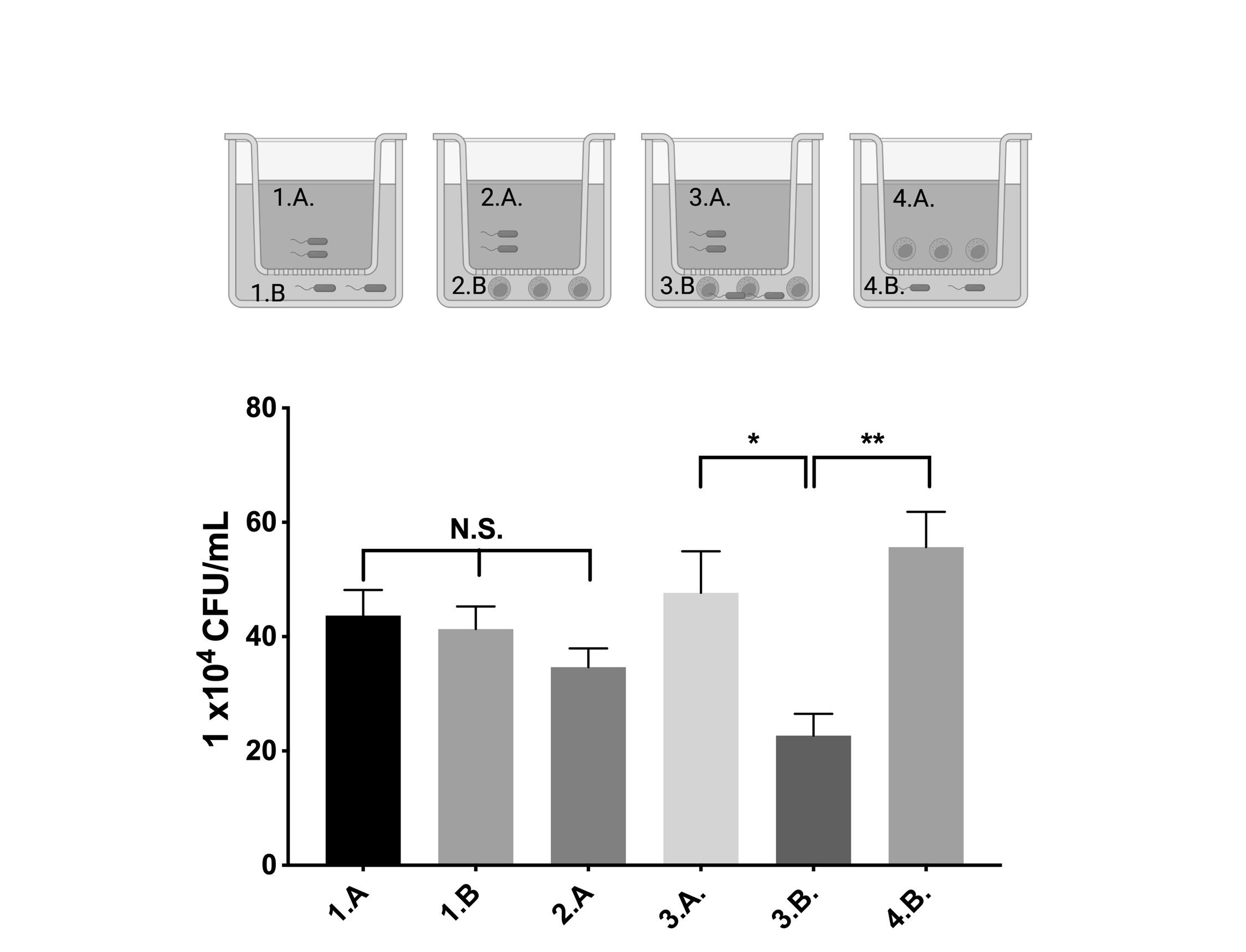

Supplement: S4 Fig — CFU of P. aeruginosa PAO1 incubated alone, separated by a 0.1um filter, or in direct contact with YT cells for 6 h. In all co-culture experiments, conditions were carried out in n = 3 wells (mean ± SEM) and the graph is representative of 2 biological replicates performed on different days. ** = P≤0.01, *** = P≤0.001. NS not significant. Illustration created by DDF using BioRender. (TIF) [file ppat.1010325.s005.tif]

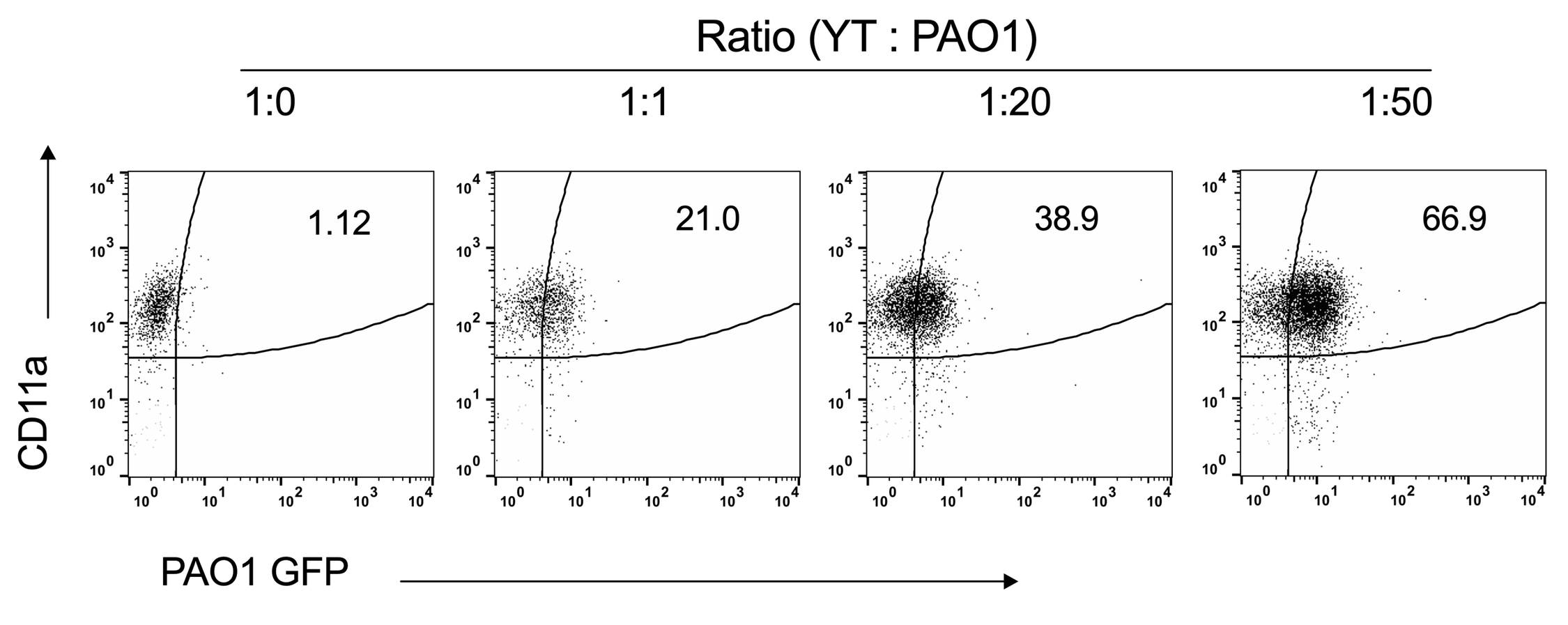

Supplement: S5 Fig — YT cells were labeled with PE-Cy5-CD11a and co-cultured with GFP-expressing PA01 for 10 minutes at the indicated ratios. Flow cytometry analysis was performed to determine the percentage of CD11a+ YT bound to GFP+ P. aeruginosa. Gating was done using YT cells, and populations that fluoresced positive for both red and green were considered conjugates, and the degree of binding was normalized to isotype control. Representative scatter plot showing the percentage of the three P. aeruginosa strains bound to YT cells. (TIF) [file ppat.1010325.s006.tif]

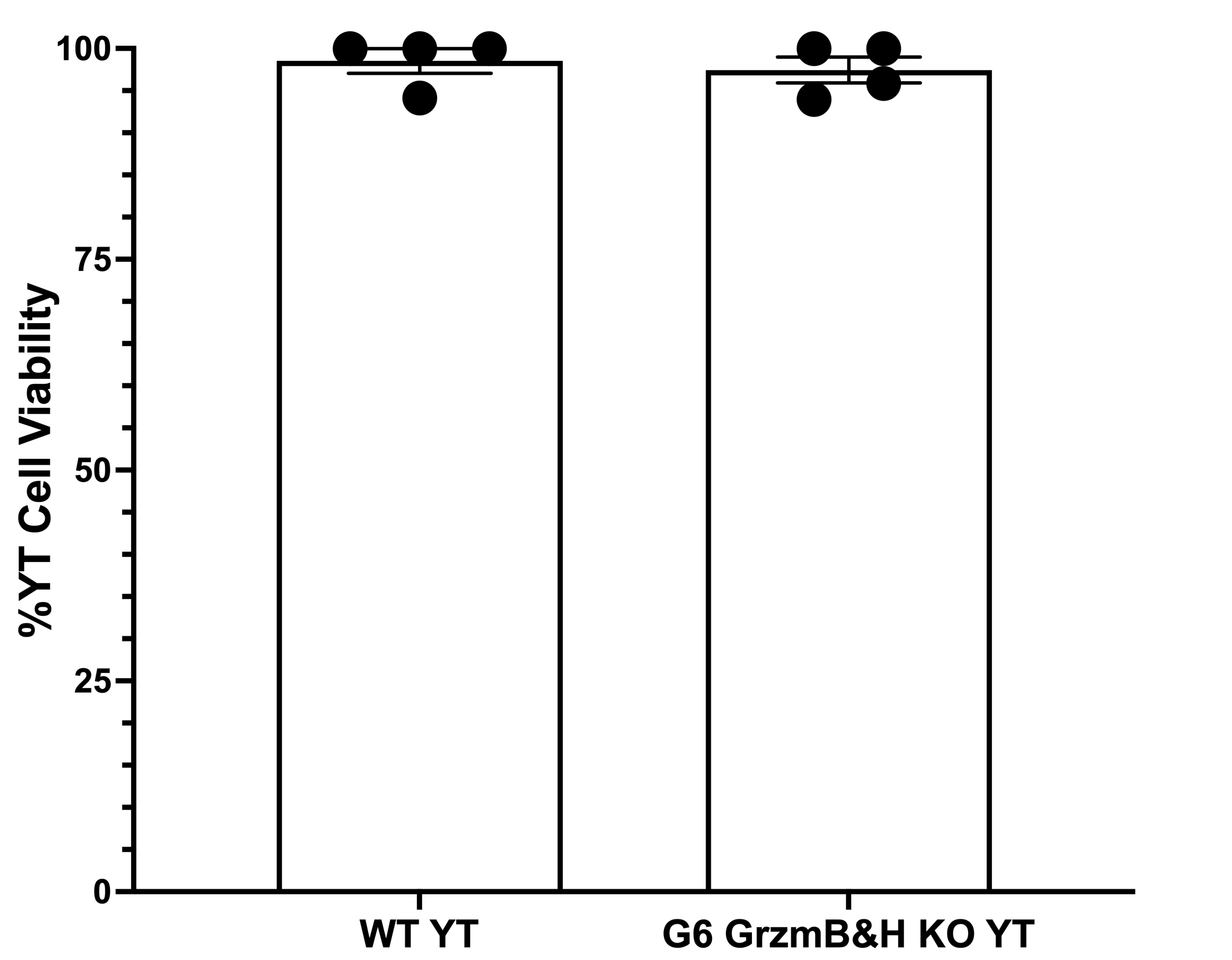

Supplement: S6 Fig — WT or GrzmB&H KO YT cells co-cultured with P. aeruginosa PAO1 for 6 h. After co-culture, the YT cell viability was assessed using trypan blue staining. Conditions were carried out in n = 4 wells (mean ± SEM) and the graph is representative of n = 1 biological replicates. (TIF) [file ppat.1010325.s007.tif]
